# Use of multidimensional item response theory methods for dementia prevalence prediction: an example using the Health and Retirement Survey and the Aging, Demographics, and Memory Study

**DOI:** 10.1186/s12911-021-01590-y

**Published:** 2021-08-11

**Authors:** GBD 2019 Dementia Collaborators, Emma Nichols, Foad Abd-Allah, Amir Abdoli, Ahmed Abualhasan, Eman Abu-Gharbieh, Ashkan Afshin, Rufus Olusola Akinyemi, Fahad Mashhour Alanezi, Vahid Alipour, Amir Almasi-Hashiani, Jalal Arabloo, Amir Ashraf-Ganjouei, Getinet Ayano, Jose L. Ayuso-Mateos, Atif Amin Baig, Maciej Banach, Miguel A. Barboza, Suzanne Lyn Barker-Collo, Bernhard T. Baune, Akshaya Srikanth Bhagavathula, Krittika Bhattacharyya, Ali Bijani, Atanu Biswas, Archith Boloor, Carol Brayne, Hermann Brenner, Katrin Burkart, Sharath Burugina Nagaraja, Felix Carvalho, Luis F. S. Castro-de-Araujo, Ferrán Catalá-López, Ester Cerin, Nicolas Cherbuin, Dinh-Toi Chu, Xiaochen Dai, Antonio Reis de Sá-Junior, Shirin Djalalinia, Abdel Douiri, David Edvardsson, Shaimaa I. El-Jaafary, Sharareh Eskandarieh, Andre Faro, Farshad Farzadfar, Valery L. Feigin, Seyed-Mohammad Fereshtehnejad, Eduarda Fernandes, Pietro Ferrara, Irina Filip, Florian Fischer, Shilpa Gaidhane, Lucia Galluzzo, Gebreamlak Gebremedhn Gebremeskel, Ahmad Ghashghaee, Alessandro Gialluisi, Elena V. Gnedovskaya, Mahaveer Golechha, Rajeev Gupta, Vladimir Hachinski, Mohammad Rifat Haider, Teklehaimanot Gereziher Haile, Mohammad Hamiduzzaman, Graeme J. Hankey, Simon I. Hay, Golnaz Heidari, Reza Heidari-Soureshjani, Hung Chak Ho, Mowafa Househ, Bing-Fang Hwang, Licia Iacoviello, Olayinka Stephen Ilesanmi, Irena M. Ilic, Milena D. Ilic, Seyed Sina Naghibi Irvani, Masao Iwagami, Ihoghosa Osamuyi Iyamu, Ravi Prakash Jha, Rizwan Kalani, André Karch, Ayele Semachew Kasa, Yousef Saleh Khader, Ejaz Ahmad Khan, Mahalaqua Nazli Khatib, Yun Jin Kim, Sezer Kisa, Adnan Kisa, Mika Kivimäki, Ai Koyanagi, Manasi Kumar, Iván Landires, Savita Lasrado, Bingyu Li, Stephen S. Lim, Xuefeng Liu, Shilpashree Madhava Kunjathur, Azeem Majeed, Preeti Malik, Man Mohan Mehndiratta, Ritesh G. Menezes, Yousef Mohammad, Salahuddin Mohammed, Ali H. Mokdad, Mohammad Ali Moni, Gabriele Nagel, Muhammad Naveed, Vinod C. Nayak, Cuong Tat Nguyen, Huong Lan Thi Nguyen, Virginia Nunez-Samudio, Andrew T. Olagunju, Samuel M. Ostroff, Nikita Otstavnov, Mayowa O. Owolabi, Fatemeh Pashazadeh Kan, Urvish K. Patel, Michael R. Phillips, Michael A. Piradov, Constance Dimity Pond, Faheem Hyder Pottoo, Sergio I. Prada, Amir Radfar, Fakher Rahim, Juwel Rana, Vahid Rashedi, Salman Rawaf, David Laith Rawaf, Nickolas Reinig, Andre M. N. Renzaho, Nima Rezaei, Aziz Rezapour, Michele Romoli, Gholamreza Roshandel, Perminder S. Sachdev, Amirhossein Sahebkar, Mohammad Ali Sahraian, Mehrnoosh Samaei, Mete Saylan, Feng Sha, Masood Ali Shaikh, Kenji Shibuya, Mika Shigematsu, Jae Il Shin, Rahman Shiri, Diego Augusto Santos Silva, Jasvinder A. Singh, Deepika Singhal, Valentin Yurievich Skryabin, Anna Aleksandrovna Skryabina, Amin Soheili, Houman Sotoudeh, Emma Elizabeth Spurlock, Cassandra E. I. Szoeke, Rafael Tabarés-Seisdedos, Biruk Wogayehu Taddele, Marcos Roberto Tovani-Palone, Gebiyaw Wudie Tsegaye, Marco Vacante, Narayanaswamy Venketasubramanian, Simone Vidale, Vasily Vlassov, Giang Thu Vu, Yuan-Pang Wang, Jordan Weiss, Abrha Hailay Weldemariam, Ronny Westerman, Anders Wimo, Andrea Sylvia Winkler, Chenkai Wu, Ali Yadollahpour, Metin Yesiltepe, Naohiro Yonemoto, Chuanhua Yu, Mikhail Sergeevich Zastrozhin, Anasthasia Zastrozhina, Zhi-Jiang Zhang, Christopher J. L. Murray, Theo Vos

**Affiliations:** grid.458416.a 0000 0004 0448 3644 MPH, IHME, 3980 15th Ave. NE, Seattle, WA 98195 USA

**Keywords:** Dementia, Prevalence, Algorithm, Validity, Global health

## Abstract

**Background:**

Data sparsity is a major limitation to estimating national and global dementia burden. Surveys with full diagnostic evaluations of dementia prevalence are prohibitively resource-intensive in many settings. However, validation samples from nationally representative surveys allow for the development of algorithms for the prediction of dementia prevalence nationally.

**Methods:**

Using cognitive testing data and data on functional limitations from Wave A (2001–2003) of the ADAMS study (*n* = 744) and the 2000 wave of the HRS study (*n* = 6358) we estimated a two-dimensional item response theory model to calculate cognition and function scores for all individuals over 70. Based on diagnostic information from the formal clinical adjudication in ADAMS, we fit a logistic regression model for the classification of dementia status using cognition and function scores and applied this algorithm to the full HRS sample to calculate dementia prevalence by age and sex.

**Results:**

Our algorithm had a cross-validated predictive accuracy of 88% (86–90), and an area under the curve of 0.97 (0.97–0.98) in ADAMS. Prevalence was higher in females than males and increased over age, with a prevalence of 4% (3–4) in individuals 70–79, 11% (9–12) in individuals 80–89 years old, and 28% (22–35) in those 90 and older.

**Conclusions:**

Our model had similar or better accuracy as compared to previously reviewed algorithms for the prediction of dementia prevalence in HRS, while utilizing more flexible methods. These methods could be more easily generalized and utilized to estimate dementia prevalence in other national surveys.

**Supplementary Information:**

The online version contains supplementary material available at 10.1186/s12911-021-01590-y.

## Background

High-quality estimates of dementia prevalence are critical for informed health system planning, especially given the high estimated prevalence of dementia, both in the United States and globally. Recent estimates suggest that there were an estimated 4.9 (95% UI 4.4–5.4) million individuals living with dementia in the United States and an estimated 51.6 (44.3–59.0) million individuals living with dementia globally in 2019 [1]. Policy- and decision-makers rely on these estimates to inform public health planning efforts and resource allocation decisions. One of the major limitations in the estimation of dementia both nationally and globally is the lack of large, nationally representative surveys with valid data on dementia prevalence using the Diagnostic and Statistical Manual (DSM) definition [2, 3]. While the lack of nationally representative data affects estimation even in high-income settings, due to the time-consuming and costly nature of dementia assessments, these limitations are especially problematic in low-income countries, where there are large data gaps and only few studies, mainly non-representative, exist [4, 5].

Many large-scale surveys, such as the Health and Retirement Survey (HRS), a nationally representative sample of older adults in the United States, do not include dementia diagnoses. However, the HRS and other similar studies conduct evaluations of cognitive ability and functional limitations, the two major determinants of dementia status [6]. The Aging, Demographics, and Memory Study (ADAMS) sampled individuals from HRS and administered an intensive diagnostic workup culminating in an adjudicated dementia diagnosis [7]. A number of algorithms have been developed for the estimation of dementia prevalence in HRS based on cut-points or regression-based methods using the ADAMS subsample and the questions on demographic information, cognitive status, and daily functional limitations that are included in both surveys [8–12]. This study aimed to improve on these methods by using multidimensional item response theory (IRT) methods to more flexibly characterize cognitive status and functional limitations, potentially facilitating the use of similar strategies in other samples.

IRT methods are used to estimate ability on an unobserved (latent) trait [13]. While the latent trait is not directly observed, answers to a series of questions (items) are used to estimate ability (a measure of an individual’s score on the latent trait) conditional on a given response pattern. IRT methods account for variations in the difficulty in the items assessed as well as variations in the strength of the relationship between different items and the latent trait. Within the IRT framework, individuals who have the same sum score (count of total correct items) can have different estimated latent cognitive abilities [14, 15].

If two different cognitive assessments have a subset of items in common, these items can be used to co-calibrate the scales so that all available information can be used and scores can be compared without sub-setting to common items [16]. While previous applications of IRT methods in epidemiology have largely focused on scoring individuals on a single, unidimensional construct, such as cognition, multidimensional IRT methods allow for the concurrent estimation of multiple, related traits [17]. The DSM criteria for dementia are based on two different but related latent traits: cognitive ability and functional (difficulty with completing daily activities) ability [18]. Therefore, a multidimensional analysis is required.

This study will improve on previous algorithms for the prediction of dementia prevalence in HRS by utilizing IRT methods to more accurately capture the cognitive and functional abilities of participants. We will also describe the potential utility of these methods for use beyond the HRS and ADAMS samples, with a focus on the benefits of their application to improve the global estimation of dementia.

## Methods

### Sample description

The Health and Retirement Study (HRS) is a nationally representative, prospective cohort study of over 37,000 individuals in the USA [6]. The study used data from the 2000 wave of the HRS survey. We excluded individuals younger than 70 years old at this wave to ensure comparability between the HRS and ADAMS samples as ADAMS was restricted to those 70 and older. The sample included 6373 individuals. The HRS study used proxy respondents to assess cognition and function when individuals were unable to complete the survey themselves (n = 1090).

The 2000 wave of HRS was used (along with the 2002 wave of HRS) as the sampling frame for the Aging, Demographics, and Memory study (ADAMS). ADAMS stratified the sampling process by age, sex, and cognitive status, sampling a larger number of individuals at the lowest levels of cognitive performance [7]. The sample included 856 individuals. Proxy respondents for each participant answered questions related to the participant’s cognitive abilities and functional limitations.

At least one non-missing response to survey items is required for the estimation of ability on a latent trait. We therefore excluded participants in both HRS and ADAMS who did not have at least one valid response on questions related to either cognition or functional limitations. In HRS, we excluded 5 individuals without data on cognition and 10 individuals without data on functional limitations yielding a final sample size of 6358. In ADAMS, we excluded 112 individuals without informant reports, and therefore without a single valid response on questions assessing functional limitations. Individuals who were excluded were not significantly different from those included in terms of their age, gender, years of education, or place of residence (nursing home vs. outside of nursing home). This exclusion led to a final sample size of 744 individuals.

### Cognitive and functional measures

HRS administered a reduced version of the Telephone Interview for Cognitive Status (TICS), which includes immediate and delayed word recall tasks, the serial 7 s subtraction task, questions of orientation to time, backwards counting, object naming, and naming the president and vice president [19]. These were supplemented with additional questions on vocabulary from the Wechsler Adult Intelligence Scale-revised (WAIS-R) vocabulary test [20]. To assess function in HRS, respondents were asked a series of questions on activities of daily living (ADLs) and instrumental activities of daily living (IADLs), which are indicative not only of physical issues but also difficulties in daily activities that may be influenced by cognition [21, 22]. When participants were unable to answer cognitive questions, the short form of Jorm’s Informant Questionnaire on Cognitive Decline in the Elderly (IQCODE) was assessed (*n* = 660) [23].

The cognitive and functional items administered in ADAMS additionally included the Consortium to Establish a Registry for Alzheimer’s Disease (CERAD) animal fluency task [24], the Mini-Mental State Examination (MMSE) [25], the three-trial CERAD word list [24], the Trail Making Test Part A and B [26], the Digit Symbol Substitution Test [27], and the Digit Span test [20]. A full table of available items in each sample is available in Additional File 1.

### Dementia adjudication

All participants in ADAMS were evaluated by a nurse and neuropsychology technician in a 3–4 h structured interview and assessment. A team of clinicians from the ADAMS study, including the study geropsychiatrist, neurologist, neuropsychologist, and cognitive neuroscientist, assigned clinical dementia diagnoses in ADAMS based on all information collected along with available medical records [28]. Diagnoses were based on DSM-III-R and DSM-IV criteria.

### Statistical analysis

**Fig. 1:**
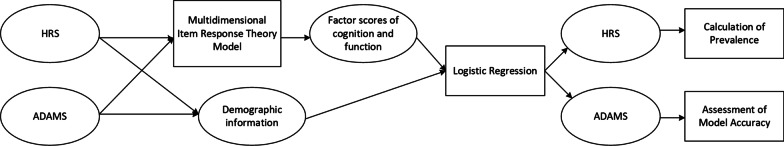
Flowchart for the estimation procedure. Data from the Health and Retirement Study (HRS) and the Aging, Demographics, and Memory study (ADAMS) are used in a multidimensional item response theory model to calculate factor scores of cognition and function. These factor scores, together with demographic information are then utilized in a logistic regression model to predict dementia status. This algorithm is then used to assess prevalence in HRS and model accuracy is assessed using ADAMS

The overall analytic strategy used in this analysis is described in Fig. 1. We first calculated descriptive statistics to compare the HRS and ADAMS samples. We used IRT methods to estimate models for latent cognitive and functional ability in both HRS and ADAMS. Items with continuous outcomes were discretized using ten category equal-interval discretization [29]. We collapsed categories until all categories contained at least 5% of the total sample to prevent instability.

Based on our a priori knowledge and the results of exploratory factor analysis models (additional details in Additional File 1), we selected a two-factor model and designated items as related to either a factor describing cognition or a factor describing physical function (full list in Additional File 1). We then estimated a multiple group two-factor IRT model using the ADAMS and HRS samples, allowing for correlation between the two factors. Within the IRT model, binary items were modelled using two-parameter logistic regression models, and ordered polytomous items were modelled using graded-response models [30]. In IRT models, individual records are assumed to be independent of each other. Therefore, individuals appearing in both the HRS 2000 wave and the ADAMS sample were excluded from the HRS sample in this model (n = 773) to ensure that only one record per individual was retained in the final model. Item parameters (loadings and thresholds) were estimated using maximum likelihood, and parameter values on items that were shared between the samples were constrained to be equal. Item loadings are a measure of the relationship between the item and the underlying latent trait on a scale of 0–1, whereas item thresholds indicate the ability level at which 50% of individuals correctly answered a binary item or endorsed a given response option for an ordinal item. We excluded items with loadings of less than 0.3, as this indicates a poor relationship with the underlying latent factor (n = 3 items). We calculated omega as a measure of the internal reliability of each of the factors estimated in our multidimensional IRT model using previously described methods [31]. Based on this model, we estimated factor scores for all participants in the two samples.

We then estimated three weighted logistic regression models to predict dementia status in the ADAMS sample accounting for the complex survey design. Our first model (base model) included only age and sex as predictors of dementia status, whereas the second model (factor score model) included only the factor scores for cognition and function. The final model (full model) included age and sex as well as the two factor scores. We compared models using the Akaike Information Criterion (AIC) and likelihood ratio tests and tested model calibration using the Hosmer–Lemeshow goodness of fit test. We evaluated model discrimination and performance using ten-fold cross validation in the ADAMS sample, and we calculated cross-validated area under the curve (AUC), sensitivity, specificity, and accuracy, defined as the proportion of individuals who were correctly classified. To derive 95% uncertainty intervals around model performance metrics, we sampled 1000 draws of predictions from our logistic regression model, accounting for uncertainty in the estimated model parameters. We then calculated performance metrics for each draw and then defined the 95% confidence interval as the 25th and 975th value of the ordered draw.

We then used the full model to predict dementia status in HRS, defining dementia as having a predicted probability greater than 0.5. To estimate dementia prevalence in HRS, we used survey weights to calculate weighted means and estimated confidence intervals accounting for the complex survey design and the variance in the sampling strategy. Statistical analysis was conducted in R, and IRT models were estimated using the mirt package [32]. R code is available via https://github.com/ihmeuw.

## Results

### Sample characteristics

The ADAMS and HRS samples both included more women than men; however, the ADAMS sample was slightly older (mean age in years; standard deviation [SD]: 81.5; 7.1) than the HRS sample (77.8; 5.8). ADAMS oversampled individuals with higher levels of cognitive impairment, and this is reflected in the lower scores on the TICS cognitive assessment, higher mean number of ADL limitations, and lower levels of education as compared to the HRS sample (Table 1).

**Table 1:** Study characteristics comparing the Health and Retirement Survey (HRS) with the Aging, Demographics and Memory Study (ADAMS)

	HRS (N = 6358)	ADAMS (N = 744)
Age: 70–79—%	70.6	46.8
Age: 80–89—%	26.6	42.1
Age: 90 + —%	2.8	11.2
Education (Less than HS)—%	34.3	50.7
Education (HS)—%	33.8	24.1
Education (More than HS)—%	31.9	25.3
Sex (Female)—%	58.2	57.9
Nursing home status (Yes)—%	0.3	9.7
TICS score—mean (SD)	9.3 (1.2)	6.8 (3.3)
Number of ADL limitations—mean (SD)	0.7 (1.3)	1.5 (2.2)

*SD* standard deviation, *HS* high school, *TICS* telephone Interview for cognitive status (score 0–10, with 10 being highest cognition), *ADL* activities of daily living (score 0–6, with 6 being needs the most help)

### IRT harmonization model

The estimated reliability coefficients for the cognition and functional limitations factors based on the multiple-group two-factor IRT model were 0.52 and 0.86 respectively. This indicates good reliability for the function scale, but points to the existence of some remaining unexplained variation in the items included in the cognition factor.

**Fig. 2:**
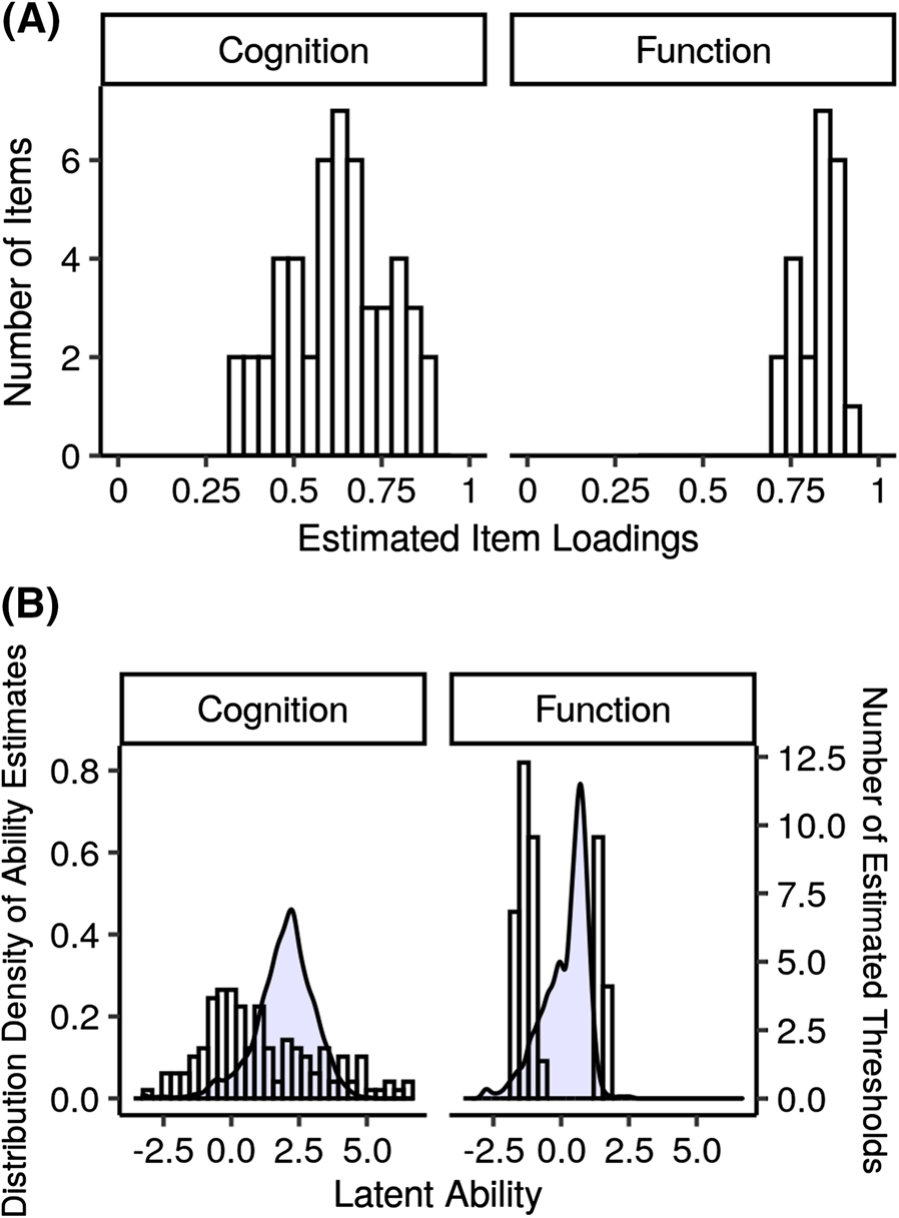
Distribution of parameters from the multidimensional item response theory model linking estimates of latent cognitive and functional ability in the Health and Retirement Study (HRS) and the Aging, Demographics, and Memory study (ADAMS). Panel (**A**) shows the distribution of item loadings. The item loadings indicate the strength of the relationship between each item and the latent trait on a scale of 0–1. Panel (**B**) shows the distribution of item thresholds and the density distribution of estimated individual-level latent cognitive and functional abilities from the two samples overlaid in blue. Thresholds indicate either the difficulty of a binary item or the difficulty of scoring one category higher on an ordinal item. A higher number of thresholds at a given estimated ability level indicates higher precision for the estimation of the latent trait at that ability level

The estimated loadings among the cognitive items ranged from 0.34 to 0.88. The items with the highest loadings were the MMSE items for naming a wristwatch, pencil, and the current year as well as the Digit Symbol Substitution Test. The loadings for the items assessing functional limitations were on average higher, ranging from 0.70 to 0.93. The items with the highest loadings were from the IQCODE proxy questionnaire and included the questions about whether the individual knew how to work familiar machines, and knew how to handle money and shop (Fig. 2; Panel A).

The estimated thresholds for cognition ranged from − 3.1 to 6.7 logits (on the scale of the latent trait) and covered the range of estimated factor scores for cognition (estimates of cognitive ability). There were a larger number of thresholds below the mean of the distribution of cognition factor scores, indicating that the questions administered were better able to precisely estimate cognition for individuals with lower levels of cognitive ability. The distribution of estimated thresholds for functional limitations was bimodal. Items assessing ADLs, IADLs, and informant reports of whether individuals declined versus retained the ability to perform a task as compared to two years ago had estimated thresholds between − 1.81 and − 0.76 logits. The second mode of thresholds spanned 1.39–1.87 logits and consisted of the informant report thresholds for whether participants improved over the last two years on everyday tasks. These thresholds correspond to a higher ability level because improving function is more difficult than preventing functional decline. The distribution of ability estimates was left-skewed, indicating that while a substantial proportion of the population did not have functional limitations, a smaller subset had a larger burden of functional limitations (Fig. 2; Panel B).

### Dementia prevalence prediction

The base model, predicting dementia status based on only age and sex, indicated that for each additional year of age, the odds of having dementia increased by 17% (95% UI 13–21). The factor score model indicated that the cognition and functional limitations each strongly predicted dementia status. For each unit increase in latent cognitive ability (a unit is one standard deviation of ability in the ADAMS sample), there was a 97% (94–98) reduction in the odds of having dementia, and for each unit increase in latent functional ability, there was a 57% (13–79) reduction in the odds of having dementia. When adjusting for age and sex in the full model, the coefficient estimates for the factor scores did not substantially change. However, the effect size for age was greatly diminished. AIC was lowest for the model that only included factor scores, and a likelihood ratio test of nested models indicated that age and sex did not improve the model once the cognitive and functional ability were accounted for (Table 2). However, we retained age and sex in our final model, because of their biological link to dementia status. The Hosmer–Lemeshow goodness of fit test indicated good calibration (*P* = 0.48) and the cross-validated area under the curve for this final model was 0.97 (0.97–0.98), indicating excellent discrimination. Based on ten-fold cross-validation in the ADAMS sample, the sensitivity of predictions from this model was 84% (80–87), the specificity of the predictions was 90% (88–92), and the overall predictive accuracy of the model was 88% (86–90).

**Table 2:** Odds ratios for the classification of dementia status in the Aging, Demographics, and Memory study (ADAMS)

	Base model	Factor score model	Full model
Age (per year)	1.17 (1.13–1.21)		1.02 (0.97–1.07)
Sex (female vs. male)	1.33 (0.83–2.14)		1.37 (0.61–3.07)
Cognition factor (per SD)		0.03 (0.02–0.06)	0.03 (0.02–0.06)
Functional limitations factor (per SD)		0.43 (0.21–0.87)	0.46 (0.23–0.92)
AIC	389.705	129.815	133.16

Odds ratios are from logistic regression models, Intervals represent 95% confidence intervals

*AIC* alkaike information criterion

**Fig. 3:**
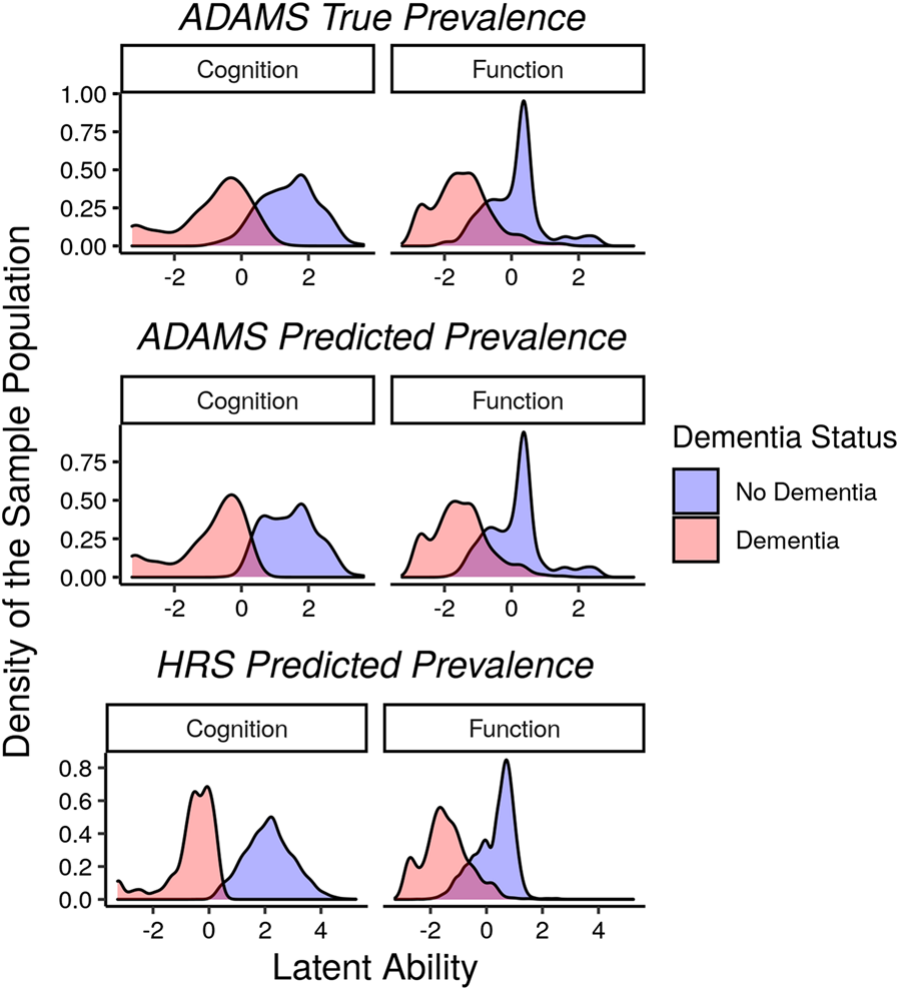
The distributions of latent cognitive and functional ability by dementia status in the Aging, Demographics, and Memory study (ADAMS) and the Health and Retirement Survey (HRS). The three plots show three different forms of dementia status: ADAMS true prevalence refers to dementia status based on the adjudicated clinician-based assessment, ADAMS predicted prevalence refers to dementia status in ADAMS based on the item response theory (IRT) algorithmic approach, and HRS predicted prevalence refers to the results of the IRT algorithmic approach in HRS

The distributions of factor scores by dementia status were similar whether classifying dementia based on true dementia status or predicted dementia status, indicating that the algorithm correctly classified most individuals. The distributions of estimated cognitive ability in the HRS sample were largely non-overlapping when comparing those classified as having versus not having dementia, indicating that the algorithm discriminates strongly on cognitive status. The overlap in the distributions was greater for functional ability as compared to cognition, due to the lack of specificity of general functional loss (Fig. 3).

**Fig. 4:**
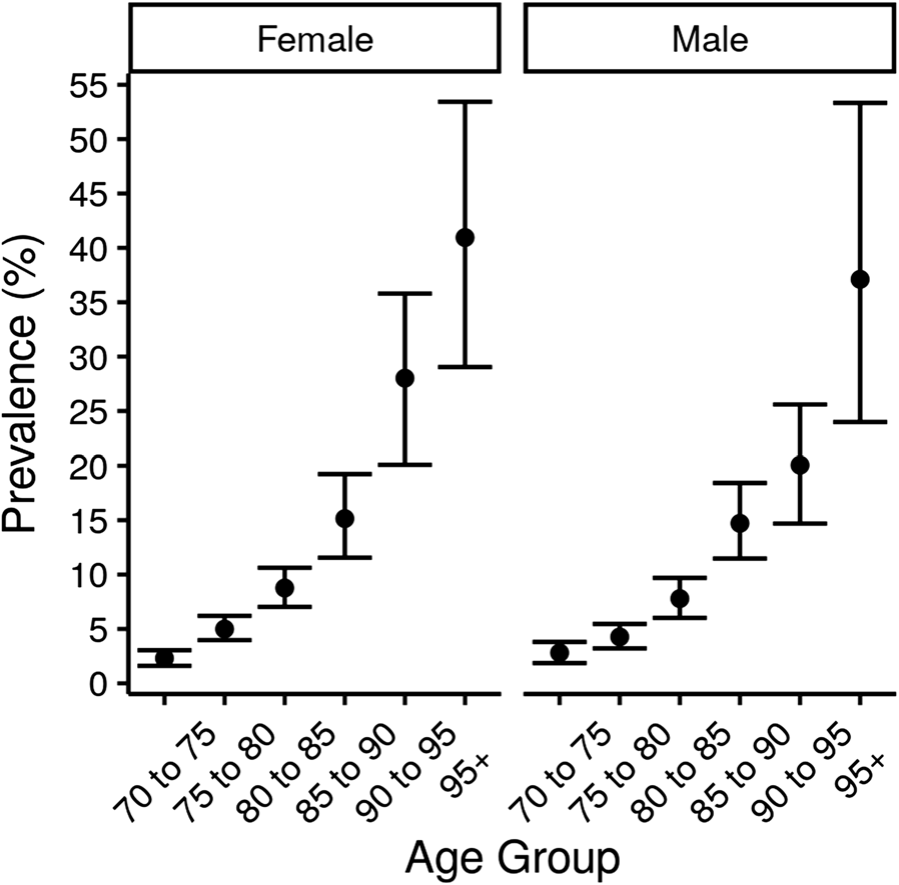
Predictions of dementia prevalence in the United States in 2000 by age and sex. These estimates were based on the application of the model developed in ADAMS to the Health and Retirement Study (HRS)

Based on the results of this model and factor scores estimated from the HRS sample, we estimated that the overall prevalence of dementia in the United States over the age of 70 was 7% (95% UI 6–7). The prevalence was higher in females than males, and prevalence increased with age, with an estimated prevalence of 4% (3–4) in individuals 70–79 years old, 11% (9–12) in individuals 80–89 years old, and 28% (22–35) in individuals 90 years old and older (Fig. 4).

## Discussion

Our algorithm had good discrimination for the classification of dementia status, with an area under the curve of 0.97 (95% UI 0.97–0.98). The model correctly classified 88% (86–90) of individuals in ADAMS, and based on the application of this model to the HRS sample, we predicted that in 2000, the prevalence of dementia over age 70 was 7% (95% UI 6–7).

In our algorithmic logistic regression models, the strong and significant effect of age was highly attenuated after accounting for cognition and functional limitations. This suggests that the effect of age was explained by the observed variation in cognition and functional limitations. Although the effect of female sex was not statistically significant, our models suggested a higher odds of dementia in women compared to men and the estimated effect size was similar to what has been previously reported in ADAMS [33].

Compared to the five algorithms reviewed in Gianattasio and colleagues (2019) and evaluated using validation data, our algorithm had the highest sensitivity, the fourth highest specificity, and the highest AUC of the regression-based algorithms [34]. Our algorithm had the same accuracy as the top-performing algorithm reviewed (the Hurd et al. algorithm, accuracy: 88%; 85–91), but this algorithm requires data on cognition from a previous wave of HRS, whereas our algorithm leverages only cross-sectional data, increasing its potential applications to settings where no longitudinal data are available [10, 34]. More recent algorithms developed with ADAMS data for use in the HRS have been shown to have similar performance as compared our algorithm in terms of sensitivity, specificity and accuracy [35]. However, our IRT-based algorithm provides a more flexible framework for algorithm development that can be leveraged to estimate prevalence in other aging surveys.

Compared to prevalence estimates from previously derived algorithms, the HRS prevalence estimates by age group derived from our algorithm (70–79 years old: 4%; 3–4, 80–89 years old: 11%; 9–12, 90+ years old: 28%; 22–35) are higher than those calculated using the Herzog-Wallace cutoffs (70–79 years old: 2%, 80–89 years old: 8%, 90+ years old: 16%) but lower than those calculated using the Langa-Weir cutoffs (70–79 years old: 8%, 80–89 years old: 20%, 90+ years old: 45%) [9]. When compared to other studies, our age-specific estimates are higher than what has been observed in the Framingham Heart Study (70–79 years old: 3%, 80–93 years old: 16%); but lower than what has been observed in the Atherosclerosis Risk in Communities Study (70–74 years old: 5%, 75–79 years old: 9%, 80–84 years old: 15%, 85–89 years old: 25%); differences between these estimates and estimates from cohort studies could be due to a number of factors including differences between the population under study and the US population as a whole [36, 37]. Our estimates are similar to what has been previously reported based on 2008 Medicare records (65–74 years old: 3%, 75–84 years old: 10%; 85+ years old: 25%) but lower than what was previously reported based only on the ADAMS subsample (70–79 years old: 5%, 80–89 years old: 24%; 9–12, 90+ years old: 37%; 22–35) [33, 38]. However, the estimates from the ADAMS sample may be biased due to the low response rate (56%) if the estimated survey weights were unable to fully correct for the patterns of non-response observed [28].

There were a number of limitations to this work. First, in ADAMS, all items on functional limitations were asked of a proxy informant, whereas items on ADLs and IADLs in HRS were administered to a proxy respondent only if the participant was unable to be interviewed. To harmonize the data on functional limitations, we assumed there would not be differences in response patterns of respondents and proxy respondents for individuals who did not have a proxy respondent in HRS as well as individuals in ADAMS who would not have needed a proxy respondent had they completed the HRS survey at that time. Previous evidence has suggested reasonable concordance between proxy-reported and self-reported activities of daily living [39, 40]. Second, while IRT methods allow for the inclusion of individuals with some missing data, we assume that among individuals who were able to complete at least some of the cognitive testing, items are missing at random [41]. However, this assumption would be violated if individuals were more likely to have missing data on the cognitive tests that were most difficult. Third, differences between the ADAMS and HRS samples could influence the performance of our algorithm, which we developed using the ADAMS sample but applied to the HRS sample. Although ADAMS participants were sampled from HRS, they were on average older and less well educated compared to the full HRS sample. Third, the response rate among individuals selected for the ADAMS sample was 56%, and this selection bias may have affected algorithm development. Although we were unable to validate our algorithm in the HRS, future work linking HRS participants with another source of information such as medical claims data would help validate the performance of the algorithm in the HRS sample. Fourth, this algorithm was developed for the purposes of overall prevalence estimation without regard to potential discrepancies by subgroups such as racial and ethnic categories. As previous work has identified biases in algorithms by racial and ethnic subgroups, this algorithm should not be applied to the study of racial subgroups without further modification [34]. Additionally, changes in the racial and ethnic composition of the US population over time may influence algorithm performance in more modern samples without algorithm re-calibration. While the ADAMS sample is a great resource for algorithm development, the sample is now 20 years old. However, the methodology developed could be used to re-calibrate the algorithm given a more current data source.

Although this algorithm is more complex than those previously developed, it is not reliant on having complete overlap in the items assessing cognition and function. Instead, all available items in both surveys can be utilized, provided there is sufficient overlap to “anchor” the scale and link scores between samples. Extending beyond the HRS sample, if a survey in another country had sufficient overlap with ADAMS on items assessing cognition and function, the ADAMS sample and the methods utilized here could be leveraged to provide prevalence estimates for surveys in other locations [14]. A number of countries (e.g., South Africa) do not have formal evaluations of dementia prevalence available but have conducted broader surveys which include evaluations of cognition and functional limitations [42]. The application of these methods to available surveys could therefore help address issues of data sparsity in the global modelling of dementia prevalence. The simplicity of the proposed algorithm, which only includes basic demographic variables in addition to cognition and functional limitations, facilitates the potential generalization of this method to the estimation of prevalence in other geographic settings. By improving data coverage and the quality of global estimates for dementia prevalence, decision-makers and policy-makers will be able to make better evidence-driven decisions around resource allocation and funding.

However, when harmonizing measures of cognition and function in the ADAMS sample with samples outside of the United Sates, it will be important to consider potential implications of differential item functioning (DIF), or differences across cultural contexts in estimated item parameters. Prior work showing evidence of DIF in cognitive items by demographic characteristics suggests that differences in cultural contexts will likely lead to some DIF, which could result in biased comparisons between samples. However, if some common items without evidence of DIF can be identified, models can be adjusted to account for DIF [43–45]. Use of smaller validation samples nested in larger surveys, such as the proposed validation sample to be conducted within the Longitudinal Aging Study in India, would allow for context-specific algorithm calibration and would circumnavigate potential concerns about DIF [46].

## Conclusions

In summary, we used multidimensional IRT-based methods to predict prevalence in the HRS sample. Compared to previous algorithms, our model had similar or better accuracy in the ADAMS sample. Furthermore, because the algorithm only relies on having a subset of items in common with a validation sample, this strategy could potentially be extended to other contexts. By improving the overall accuracy of predictive algorithms and potentially allowing researchers to leverage new data sources, this algorithmic strategy could serve to strengthen national and global estimates of dementia and improve the evidence on which policy-makers can base important decisions surrounding public health planning efforts and the resource allocation.

## Supplementary Information

**Additional file 1**: Appendix—the appendix contains supplementary information on the methodology used, and also contains some additional figures and results.

**Additional file 2**: Author names, affiliations and email addresses—this file contains all the information for authors included as part of the GBD 2019 Dementia Collaborators.

## Data Availability

The data underlying this article were provided by the Institute for Social Research at the University of Michigan. Data can be accessed by applying through the Health and Retirement Study at https://hrs.isr.umich.edu/.

## Abbreviations

ADAMS: Aging Demographics and Memory Study
HRS: Health and Retirement Study
DSM: Diagnostic and statistical manual
IRT: Item response theory
TICS: Telephone interview for cognitive status
WAIS-R: Wechsler adult intelligence scale-revised
ADL: Activities of daily living
IADL: Instrumental activities of daily living
IQCODE: Informant Questionnaire on Cognitive Decline in the Elderly
CERAD: Consortium to Establish a Registry for Alzheimer’s Disease
MMSE: Mini-Mental State Examination
AIC: Akaike information criterion
AUC: Area under the curve
SD: Standard deviation
DIF: Differential item functioning
