# Supplementary material for: Use of multidimensional item response theory methods for dementia prevalence prediction: an example using the Health and Retirement Survey and the Aging, Demographics, and Memory Study
Source: BMC Med Inform Decis Mak. 2021 Aug 11;21:241. doi: 10.1186/s12911-021-01590-y (PMC8356410; doi:10.1186/s12911-021-01590-y)
Supplement: Supplementary file 1 — Additional file 1: Appendix—the appendix contains supplementary information on the methodology used, and also contains some additional figures and results. [file 12911_2021_1590_MOESM1_ESM.pdf]

Appendix

Contents

Items included from the Health and Retirement Study (HRS) and the Aging Demographics and Memory Study (ADAMS) 2

Diagnoses Included under the Category of Dementia in ADAMS 3

Scree Plots to Assess Dimensionality 4

Table of Standardized Item Loadings 5

Multidimensional Item Response Theory Model Parameter Graph 7

Item Characteristic Curves for All Items Included 8

Cross-validated ROC Curve for Algorithm Performance in ADAMS 14

Items included from the Health and Retirement Study (HRS) and the Aging Demographics and Memory Study (ADAMS)

Item	ADAMS	HRS
ADLs	X	X
IADLs	X	X
TICS	X	X
Jorm IQCODE	X	X (only when proxy needed)
Immediate Word Recall	X (3 Trials)	X (1 trial)
Delayed Word Recall	X	X
MMSE Orientation (Year, Date, Day of Week)	X	X
MMSE (All Other)	X	
Vocabulary Items		X
Trail Making Test (A & B)	X	
Digits Span (Forward and Backwards)	X	
Symbol Digit Substitution Test	X	
Category Fluency – Animals (CERAD)	X	

*Vocabulary Test in HRS included questions asking the meaning of the words enormous, perimeter, remorse, plagiarize and audacious

** ADL = Activities of Daily Living, IADL = Instrumental Activities of Daily Living, IQCODE = Informant Questionnaire on Cognitive Decline in the Elderly, MMSE = Mini-Mental State Examination

Diagnoses Included under the Category of Dementia in ADAMS

- Probable/possible Alzheimer’s disease
- Probable/possible vascular dementia
- Normal-pressure hydrocephalus
- Dementia of undetermined aetiology
- Pick’s disease
- Frontal lobe dementia
- Alcoholic dementia
- Amyotrophic lateral sclerosis with dementia
- Hypoperfusion dementia
- Probable Lewy body dementia
- Post-encephalitic dementia

Scree Plots to Assess Dimensionality

Exploratory factor analysis and scree plots were used to assess the number of factors that exist in the data using a data-driven approach. We used an oblimin rotation to calculate eigenvalues, as this allows factors to be correlated and we believed a priori that cognition and functional limitations would be correlated. The eigenvalues represent the amount of the total variance that can be explained by a given factor. In this graph, each panel represents a different exploratory factor analysis model, with a different number of factors specified and each dot represents a factor in the exploratory factor analysis model. Factors to the left of the “elbow” of the graph, or the point at which the eigenvalues level off, are considered “important” and should be retained in the model, as these factors explain the majority of the variance in the data. In the most models, the first two factors explain the majority of the variance.

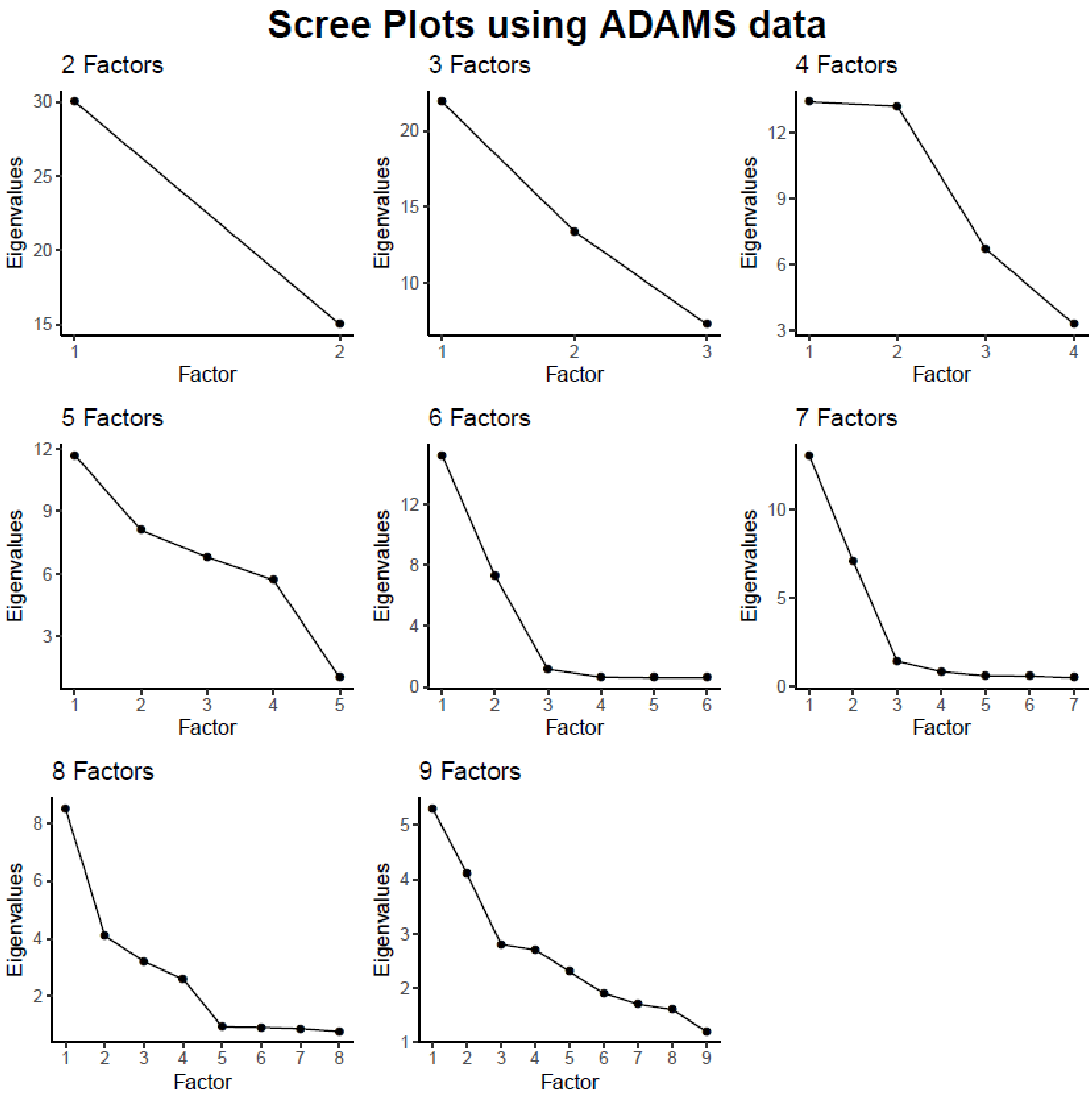

Table of Standardized Item Loadings

Cognition		Function	
**Item**	**Loading**	**Item**	**Loading**
MMSE (16)	0.88	IQCODE (math)	0.93
Orientation: Year	0.86	IQCODE (shopping)	0.9
DSST	0.84	IQCODE (machine)	0.89
TMT-B	0.84	IADL (shopping)	0.88
Immediate Word Recall (2)	0.82	IQCODE (money)	0.88
MMSE (17)	0.82	IQCODE (decisions)	0.88
Immediate Word Recall (1)	0.8	IQCODE (intelligence)	0.87
Immediate Word Recall (3)	0.8	IADL (money)	0.85
Orientation: Building Location	0.79	IALD (medications - if needed)	0.85
Orientation: Address	0.77	IADL (cooking)	0.85
Oreintation: Month	0.75	IQCODE (learn)	0.85
Category Fluency - Animals	0.74	IQCODE (gadget)	0.84
Orientation: Town	0.72	IADL (phone)	0.83
Orientation: Day of Week	0.72	ADL (bathing)	0.82
Digit Span Backwards	0.71	IQCODE (stories)	0.82
Delayed Word Recall	0.69	ADL (eating)	0.79
Oreintation: County	0.69	ADL (walking)	0.77
Orientation: Floor	0.69	IQCODE (where things kept)	0.77
TICS: President	0.69	ADL (transferring)	0.75
MMSE (11)	0.68	ADL (dressing)	0.74
MMSE (12)	0.66	IADL (meds)	0.71
Digit Span Forward	0.64	ADL (toileting)	0.7
MMSE (19)	0.64		
MMSE (21)	0.64		
MMSE (13)	0.64		
TICS: Vice president	0.64		
TICS: Cut paper	0.63		
Orientation: Season	0.62		
MMSE (15)	0.61		
MMSE (22)	0.6		
MMSE (14)	0.6		
Backwards count (20)	0.6		
TICS: Desert plant	0.57		
TMT-A	0.57		
IQCODE (date)	0.54		
IQCODE (address)	0.53		
Backwards count (86)	0.53		
IQCODE (family)	0.52		

Serial 7s	0.52	
IQCODE (recent event)	0.51	
IQCODE (conversation)	0.48	
MMSE (18)	0.47	
MMSE (20)	0.47	
Orientation: Date	0.45	
IQCODE (different place)	0.43	
Vocab (plagiarize)	0.41	
Vocab (remorse)	0.39	
Vocab (audacious)	0.36	
Vocab (enormous)	0.35	
Vocab (perimeter)	0.34	

* Numbers in parentheses after MMSE items refer to the item number of the MMSE

** Numbers in parentheses after word recall items refers to the trial number

Multidimensional Item Response Theory Model Parameter Graph

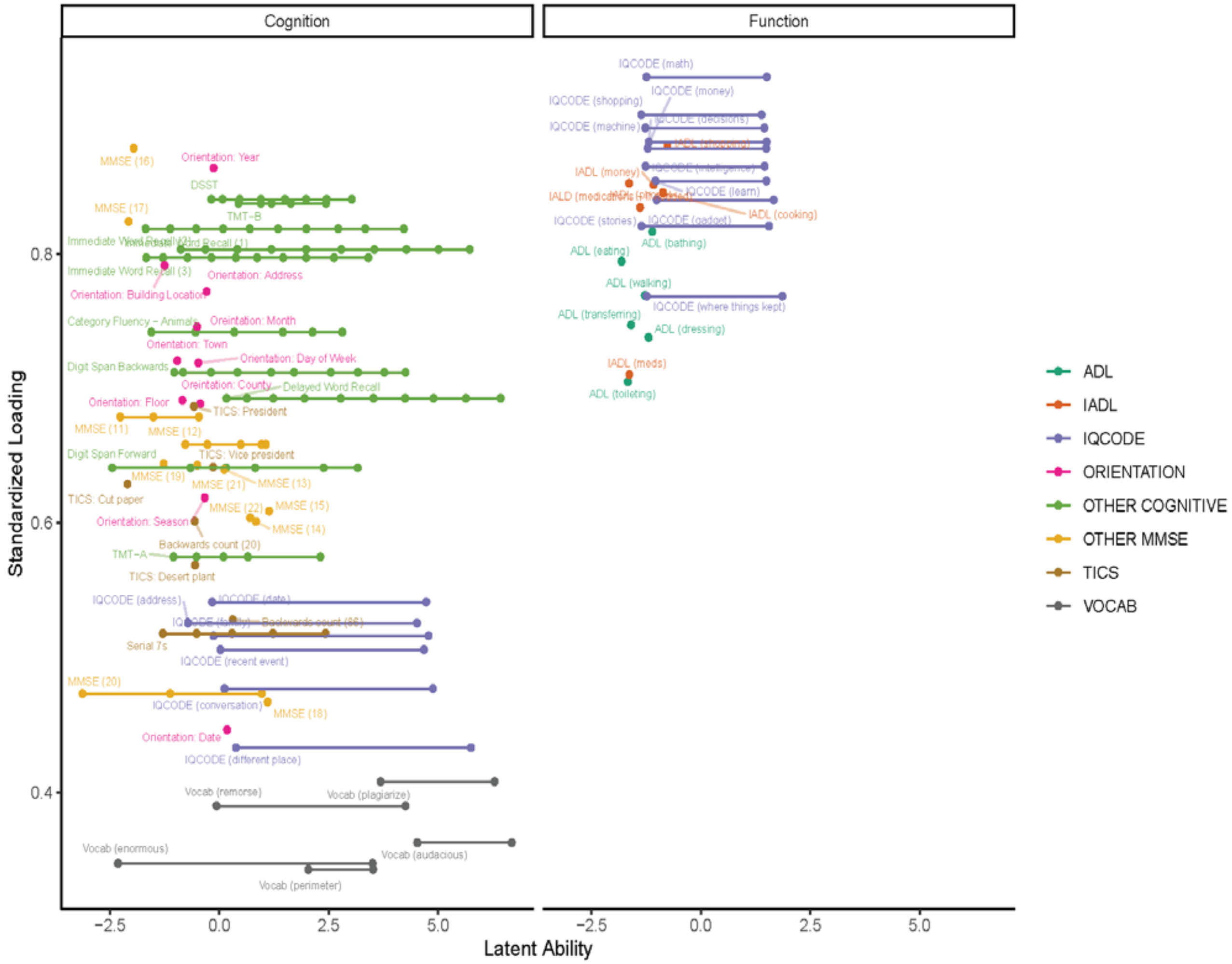

Each dot or connected line of dots represents a question asked in the Health and Retirement Study or the Aging, Demographics and Memory Study. The position of each dot on the x axis represents a threshold of that item, or the estimated difficulty of each item or additional point on an ordinal scale. The position of the dot or line on the y axis represents the standardized loading (on a scale of 0 to 1), which is a measure of the strength of the relationship between the item and the underlying trait.

Item Characteristic Curves for All Items Included

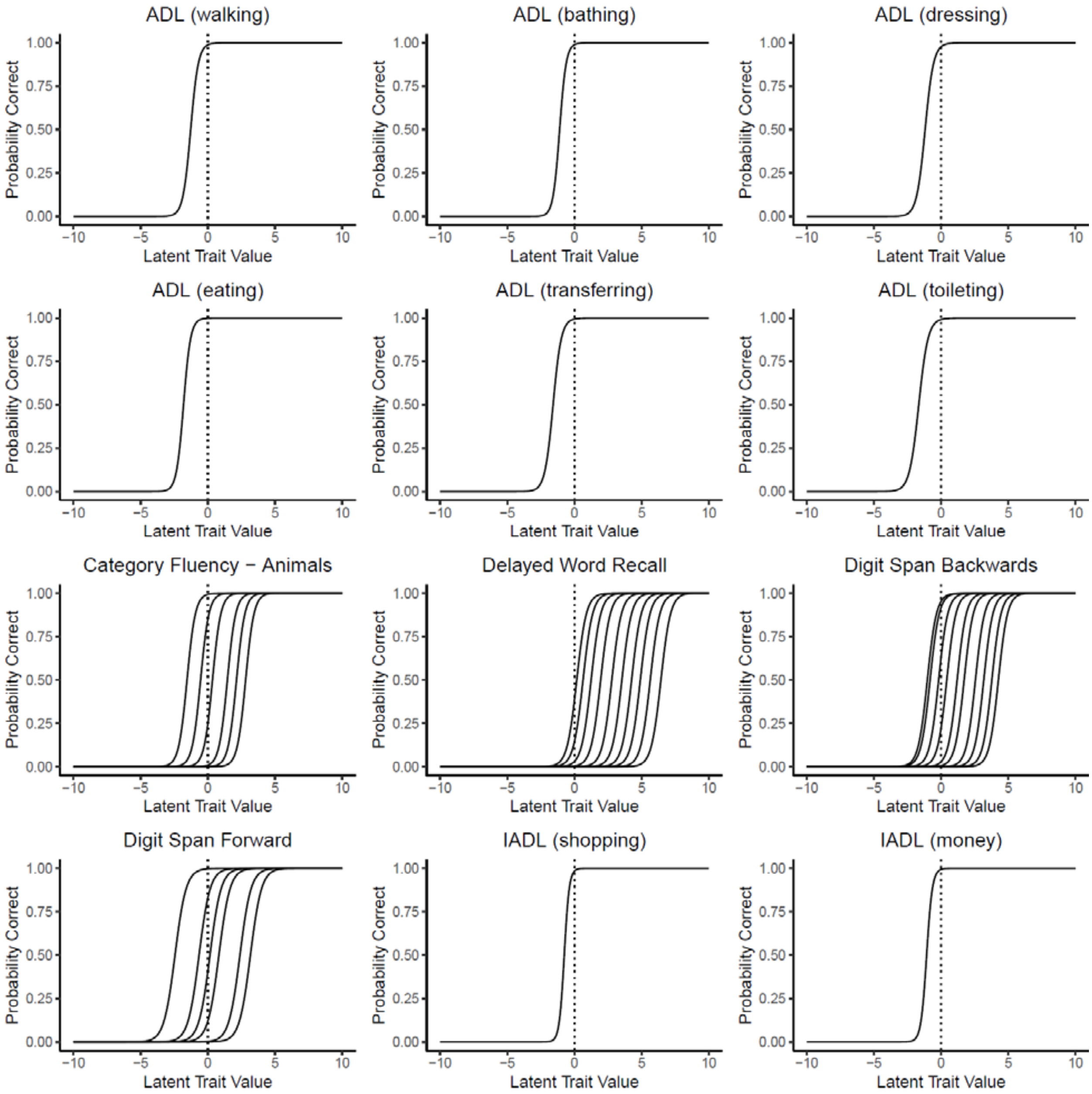

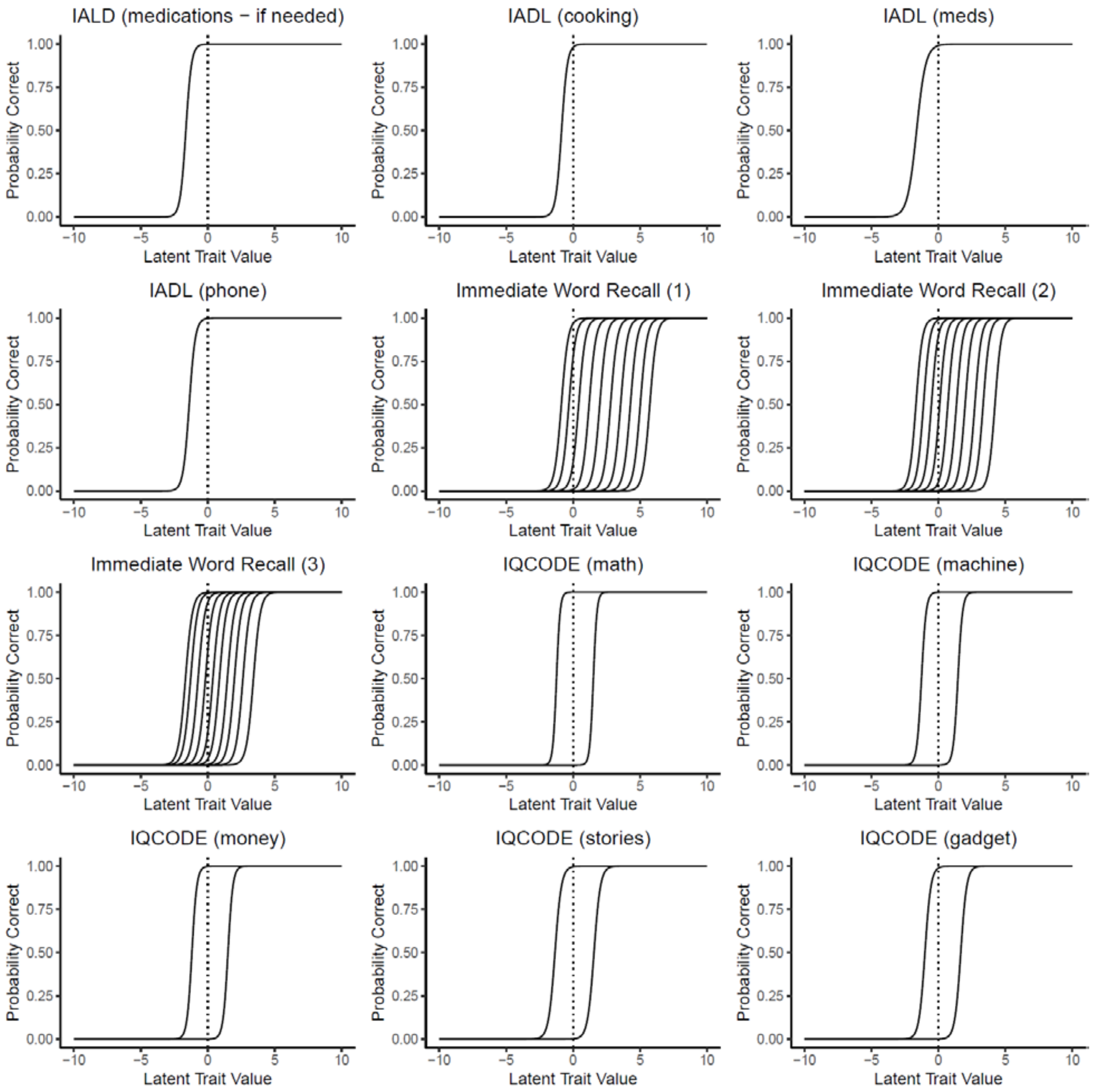
IALD (medications − if needed)
IADL (cooking)
IADL (meds)
IADL (phone)
Immediate Word Recall (1)
Immediate Word Recall (2)
Immediate Word Recall (3)
IQCODE (math)
IQCODE (machine)
IQCODE (money)
IQCODE (stories)
IQCODE (gadget)
Probability Correct
Latent Trait Value
0.00
0.25
0.50
0.75
1.00
−10
−5
0
5
10

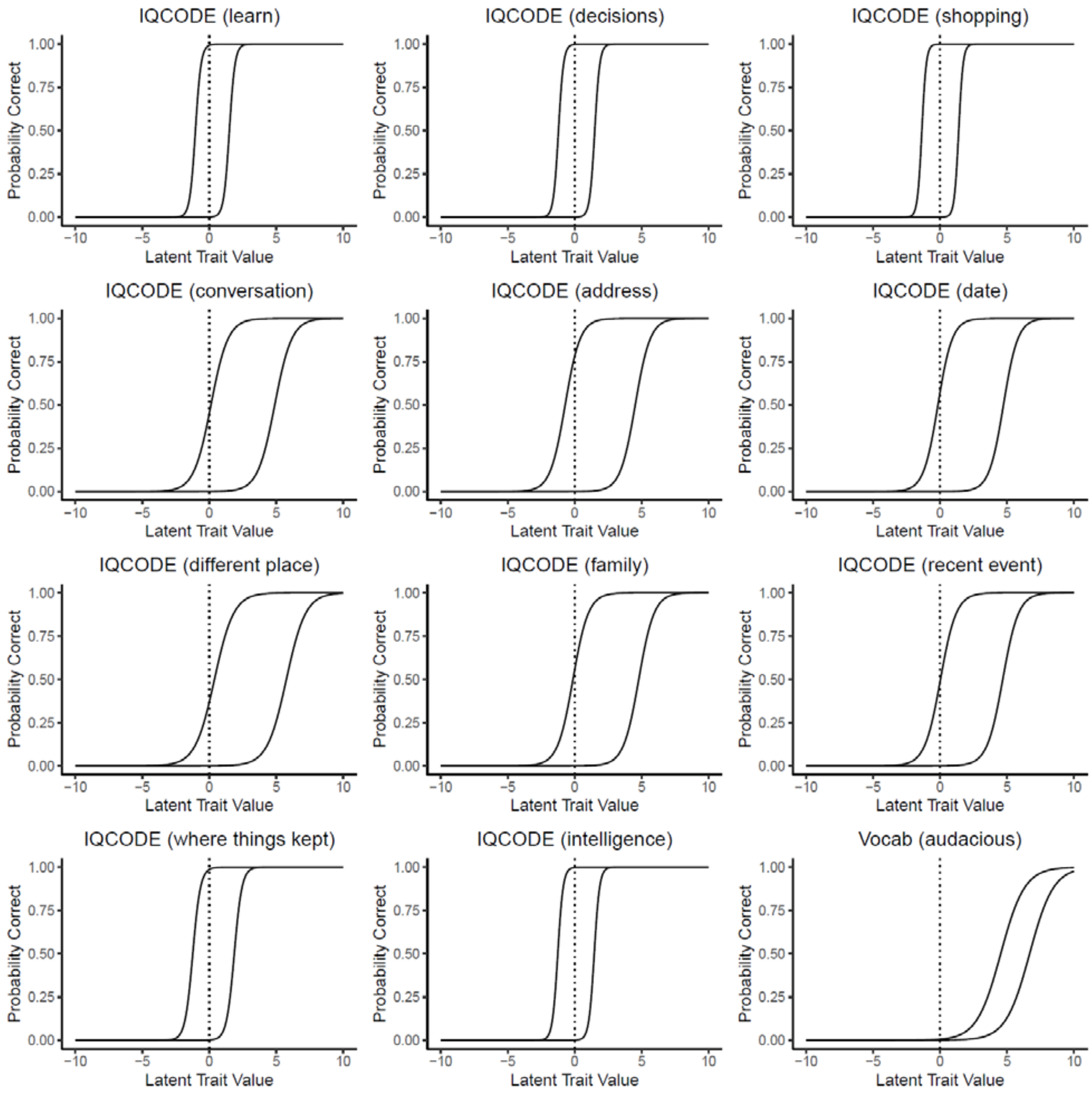

IQCODE (learn)
IQCODE (decisions)
IQCODE (shopping)
IQCODE (conversation)
IQCODE (address)
IQCODE (date)
IQCODE (different place)
IQCODE (family)
IQCODE (recent event)
IQCODE (where things kept)
IQCODE (intelligence)
Vocab (audacious)
Probability Correct
Latent Trait Value

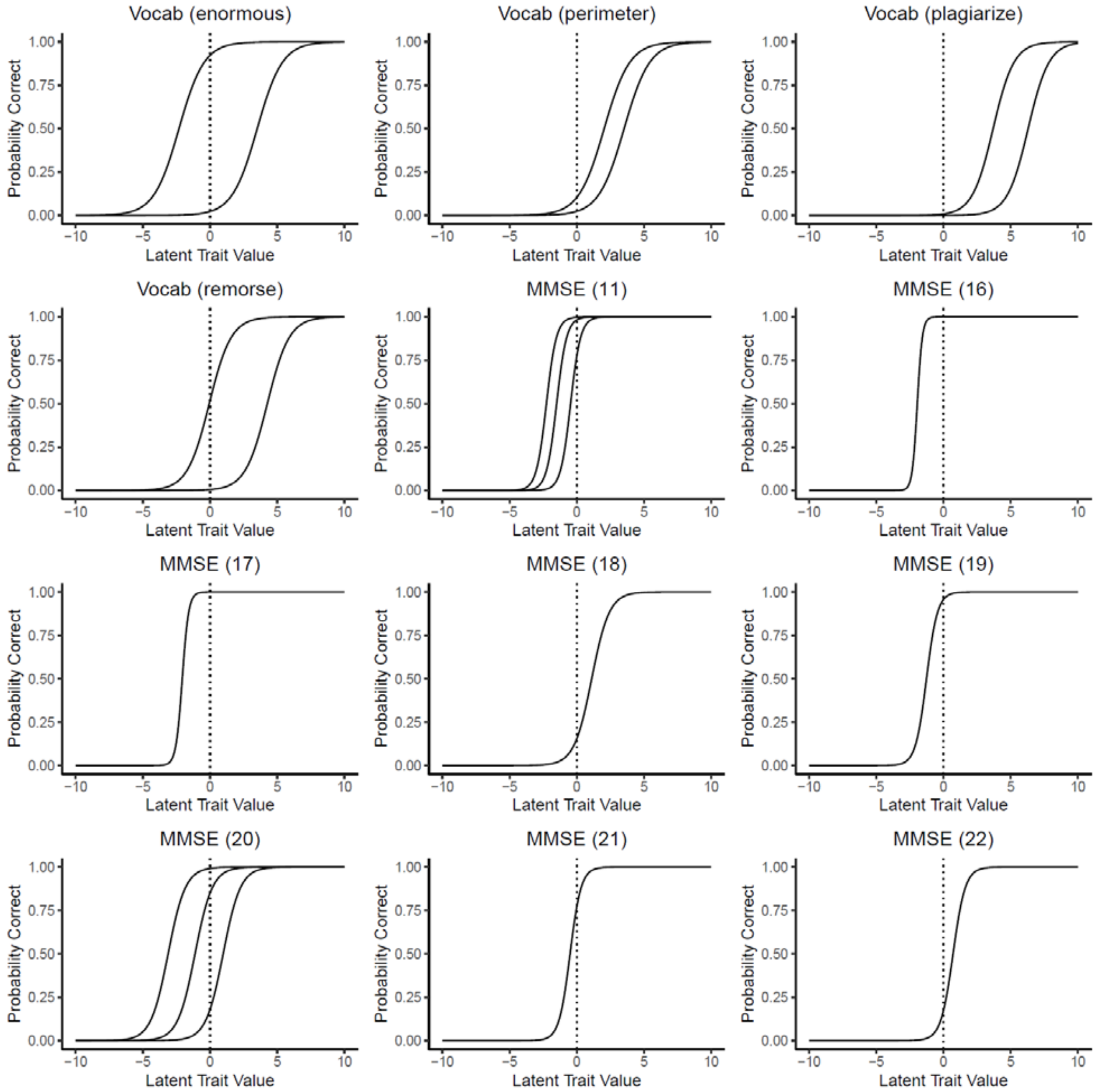
Vocab (enormous)
Vocab (perimeter)
Vocab (plagiarize)
Vocab (remorse)
MMSE (11)
MMSE (16)
MMSE (17)
MMSE (18)
MMSE (19)
MMSE (20)
MMSE (21)
MMSE (22)
Probability Correct
Latent Trait Value
1.00
0.75
0.50
0.25
0.00
−10
−5
0
5
10

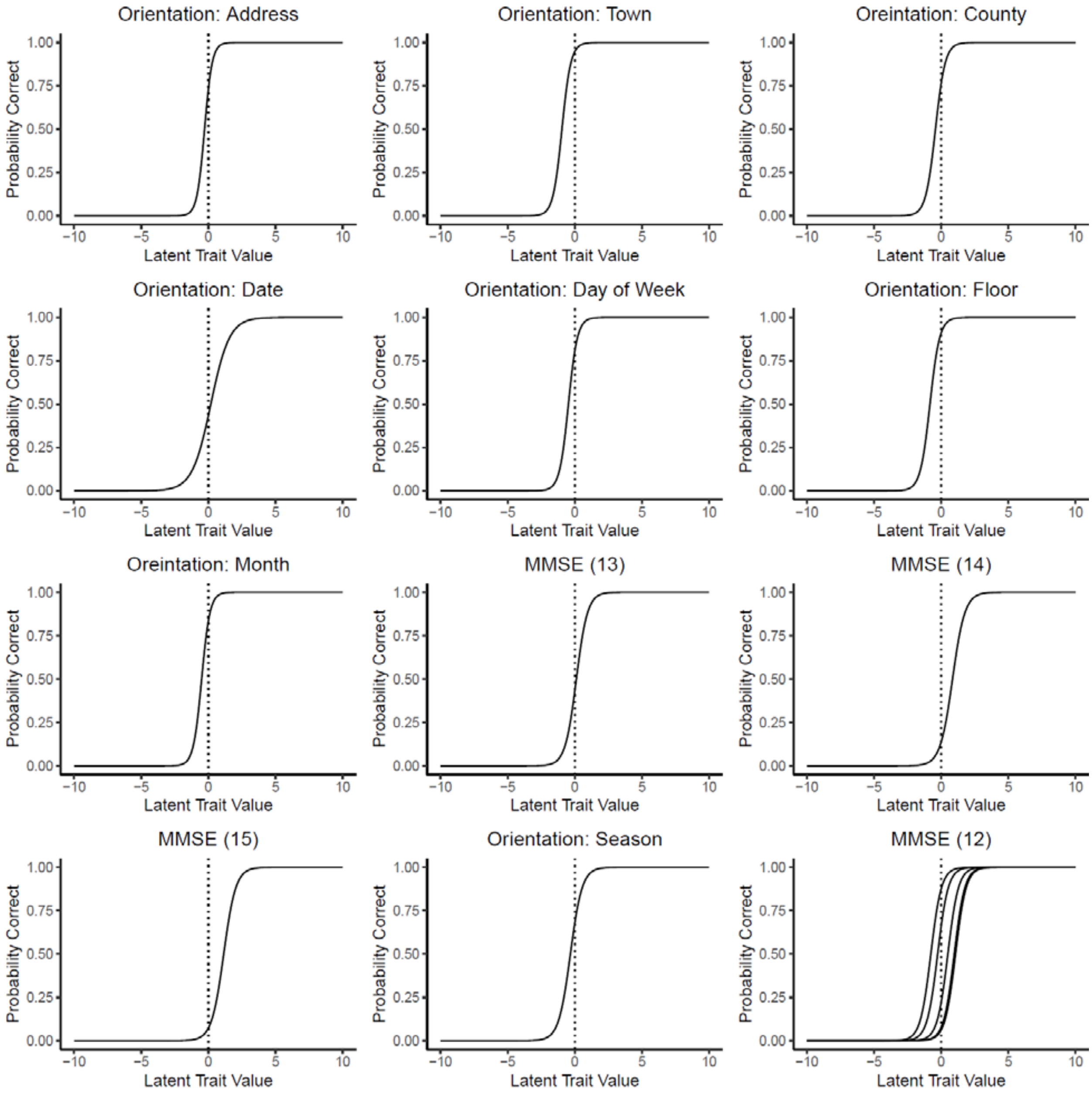

Orientation: Address
Orientation: Town
Oreintation: County
Orientation: Date
Orientation: Day of Week
Orientation: Floor
Oreintation: Month
MMSE (13)
MMSE (14)
MMSE (15)
Orientation: Season
MMSE (12)
Probability Correct
Latent Trait Value
0.00
0.25
0.50
0.75
1.00
−10
−5
0
5
10

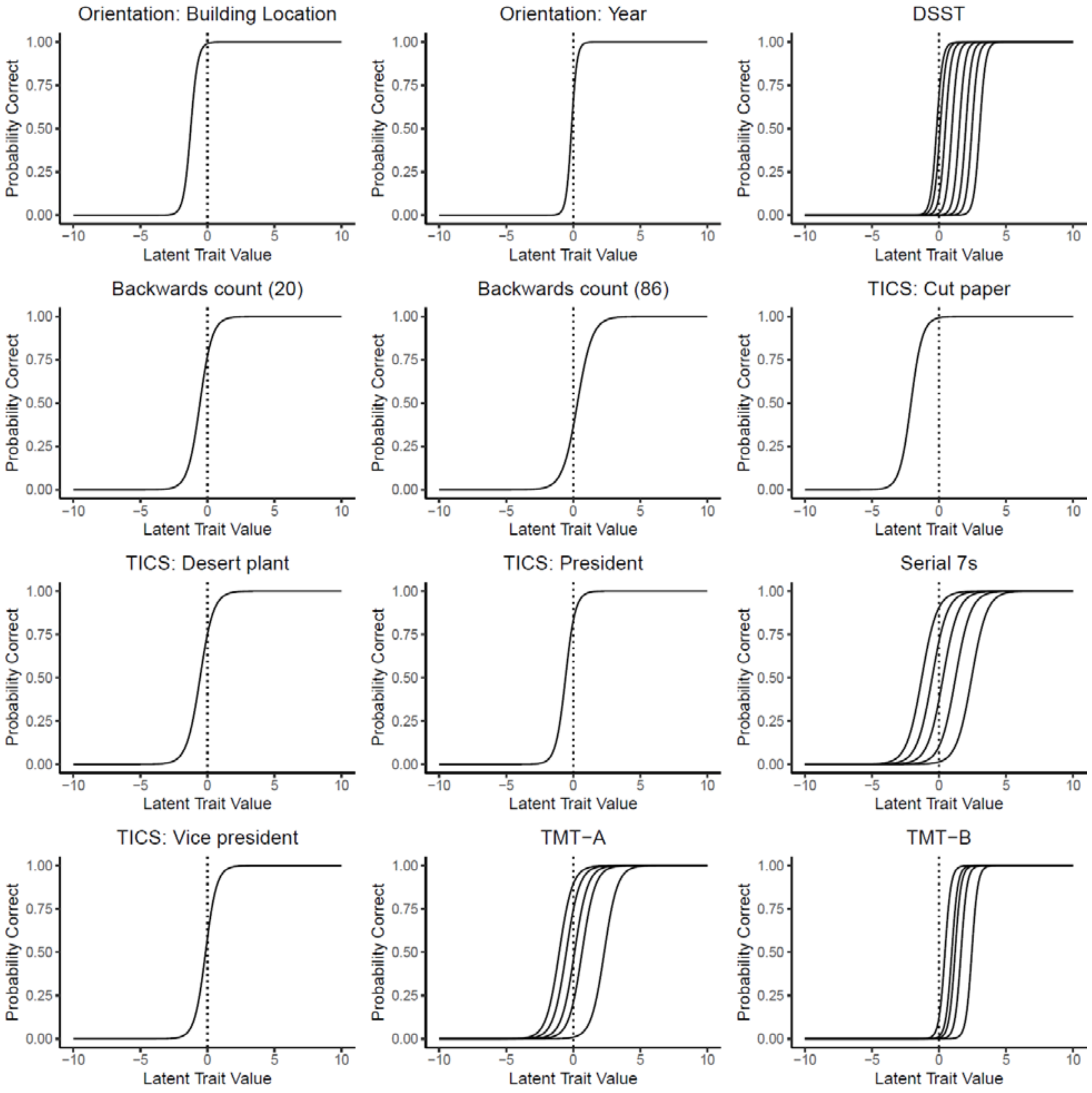
Orientation: Building Location
Orientation: Year
DSST
Backwards count (20)
Backwards count (86)
TICS: Cut paper
TICS: Desert plant
TICS: President
Serial 7s
TICS: Vice president
TMT−A
TMT−B
Probability Correct
Latent Trait Value

Cross-validated ROC Curve for Algorithm Performance in ADAMS

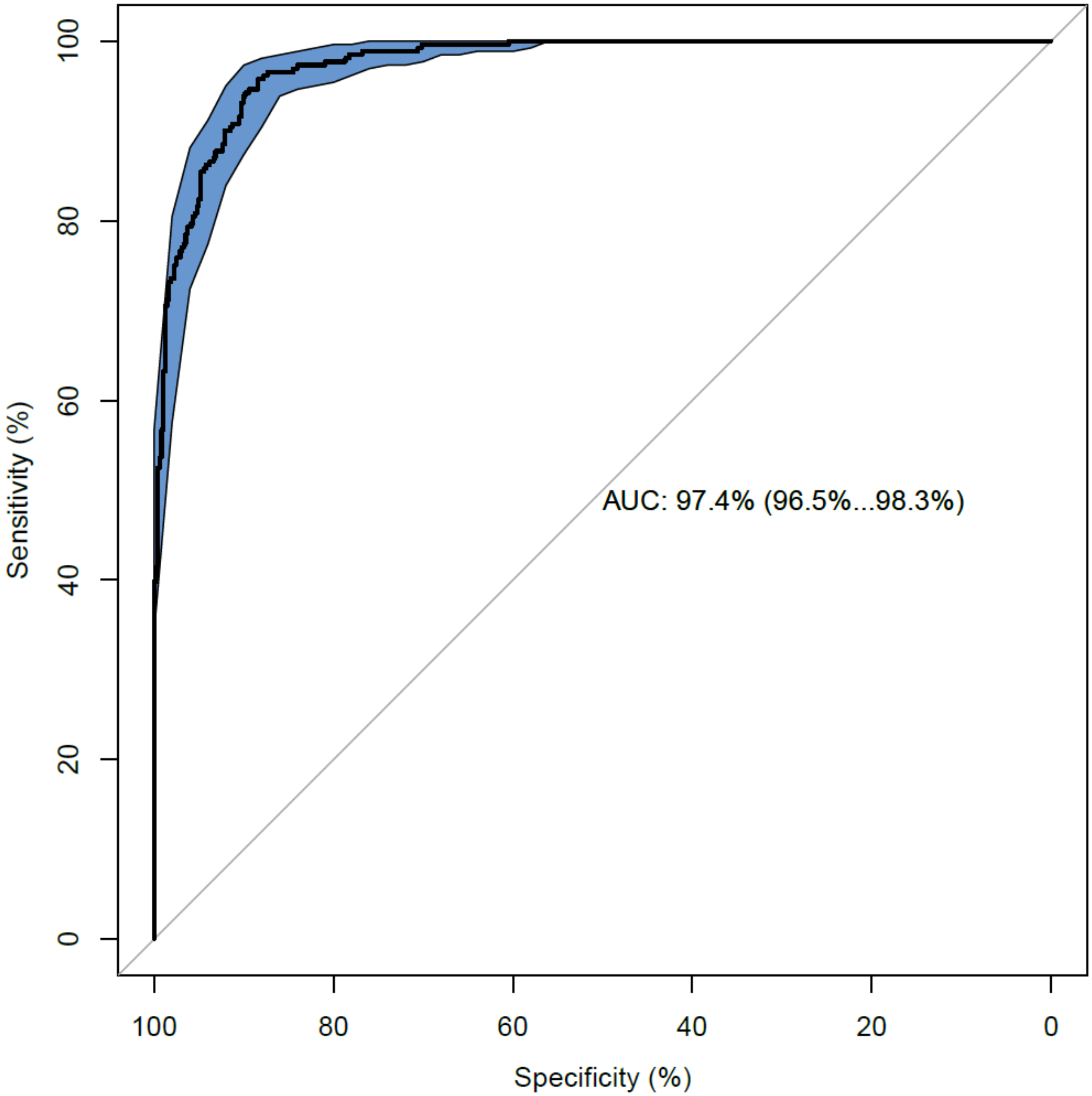

The cross-validated ROC curve indicates excellent discrimination of the multidimensional item response theory algorithm. AUC = Area under the curve.
