# Supplementary material for: Use of multidimensional item response theory methods for dementia prevalence prediction: an example using the Health and Retirement Survey and the Aging, Demographics, and Memory Study
Source: BMC Med Inform Decis Mak. 2021 Aug 11;21:241. doi: 10.1186/s12911-021-01590-y (PMC8356410; doi:10.1186/s12911-021-01590-y)
Supplement: Supplementary file 2 — Additional file 2: Author names, affiliations and email addresses—this file contains all the information for authors included as part of the GBD 2019 Dementia Collaborators. [file 12911_2021_1590_MOESM2_ESM.docx]

Emma Nichols*,^1^ Prof. Foad Abd-Allah,^2^ Amir Abdoli,^3^ Ahmed Abualhasan,^2^ Eman Abu-Gharbieh,^4^ Ashkan Afshin,^1,5^ Rufus Olusola Akinyemi,^6,7^ Fahad Mashhour Alanezi,^8^ Vahid Alipour,^9,10^ Amir Almasi-Hashiani,^11^ Jalal Arabloo,^9^ Amir Ashraf-Ganjouei,^12^ Getinet Ayano,^13^ Prof. Jose L Ayuso-Mateos,^14,15^ Atif Amin Baig,^16^ Prof. Maciej Banach,^17,18^ Prof. Miguel A Barboza,^19,20^ Prof. Suzanne Lyn Barker-Collo,^21^ Prof. Bernhard T Baune,^22,23^ Akshaya Srikanth Bhagavathula,^24,25^ Krittika Bhattacharyya,^26,27^ Ali Bijani,^28^ Prof. Atanu Biswas,^29^ Archith Boloor,^30^ Prof. Carol Brayne,^31^ Prof. Hermann Brenner,^32^ Katrin Burkart,^1,5^ Prof. Sharath Burugina Nagaraja,^33^ Prof. Felix Carvalho,^34^ Luis F S Castro-de-Araujo,^35^ Ferrán Catalá-López,^36,37^ Prof. Ester Cerin,^38,39^ Prof. Nicolas Cherbuin,^40^ Dinh-Toi Chu,^41^ Xiaochen Dai,^1^ Prof. Antonio Reis de Sá-Junior,^42^ Shirin Djalalinia,^43^ Abdel Douiri,^44^ Prof. David Edvardsson,^45,46^ Shaimaa I El-Jaafary,^2^ Sharareh Eskandarieh,^47^ Prof. Andre Faro,^48^ Prof. Farshad Farzadfar,^49^ Prof. Valery L Feigin,^50,1,51,52^ Seyed-Mohammad Fereshtehnejad,^53,54^ Prof. Eduarda Fernandes,^55^ Pietro Ferrara,^56^ Irina Filip,^57,58^ Florian Fischer,^59^ Shilpa Gaidhane,^60^ Lucia Galluzzo,^61^ Gebreamlak Gebremedhn Gebremeskel,^62,63^ Ahmad Ghashghaee,^9,64^ Alessandro Gialluisi,^65^ Elena V Gnedovskaya,^66^ Mahaveer Golechha,^67^ Prof. Rajeev Gupta,^68,69^ Vladimir Hachinski,^70,71^ Mohammad Rifat Haider,^72^ Teklehaimanot Gereziher Haile,^62^ Mohammad Hamiduzzaman,^73^ Prof. Graeme J Hankey,^74,75^ Prof. Simon I Hay,^1,5^ Golnaz Heidari,^76^ Reza Heidari-Soureshjani,^77^ Hung Chak Ho,^78^ Prof. Mowafa Househ,^79^ Prof. Bing-Fang Hwang,^80^ Prof. Licia Iacoviello,^65,81^ Olayinka Stephen Ilesanmi,^82,83^ Irena M Ilic,^84^ Prof. Milena D Ilic,^85^ Seyed Sina Naghibi Irvani,^86^ Masao Iwagami,^87,88^ Ihoghosa Osamuyi Iyamu,^89,90^ Ravi Prakash Jha,^91,92^ Rizwan Kalani,^93^ André Karch,^94^ Ayele Semachew Kasa,^95^ Prof. Yousef Saleh Khader,^96^ Ejaz Ahmad Khan,^97^ Prof. Mahalaqua Nazli Khatib,^98^ Yun Jin Kim,^99^ Sezer Kisa,^100^ Prof. Adnan Kisa,^101,102^ Prof. Mika Kivimäki,^103,104^ Ai Koyanagi,^105,106^ Manasi Kumar,^107,108^ Prof. Iván Landires,^109,110^ Savita Lasrado,^111^ Bingyu Li,^112^ Prof. Stephen S Lim,^1,5^ Xuefeng Liu,^113^ Shilpashree Madhava Kunjathur,^114^ Prof. Azeem Majeed,^115^ Preeti Malik,^116,117^ Prof. Man Mohan Mehndiratta,^118,119^ Prof. Ritesh G Menezes,^120^ Yousef Mohammad,^121^ Salahuddin Mohammed,^122,123^ Prof. Ali H Mokdad,^1,5^ Mohammad Ali Moni,^124^ Prof. Gabriele Nagel,^125^ Dr Muhammad Naveed,^126^ Prof. Vinod C Nayak,^127^ Cuong Tat Nguyen,^128^ Huong Lan Thi Nguyen,^128^ Virginia Nunez-Samudio,^129,130^ Andrew T Olagunju,^131,132^ Samuel M Ostroff,^1,133^ Nikita Otstavnov,^134^ Prof. Mayowa O Owolabi,^135,136^ Fatemeh Pashazadeh Kan,^137^ Urvish K Patel,^138^ Prof. Michael R Phillips,^139,140^ Prof. Michael A Piradov,^141^ Prof. Constance Dimity Pond,^142^ Faheem Hyder Pottoo,^143^ Sergio I Prada,^144,145^ Amir Radfar,^146^ Fakher Rahim,^147,148^ Juwel Rana,^149,150^ Vahid Rashedi,^151^ Prof. Salman Rawaf,^115,152^ David Laith Rawaf,^153,154^ Nickolas Reinig,^1^ Prof. Andre M N Renzaho,^155,156^ Prof. Nima Rezaei,^157,158^ Aziz Rezapour,^9^ Michele Romoli,^159,160^ Gholamreza Roshandel,^161^ Prof. Perminder S Sachdev,^162,163^ Amirhossein Sahebkar,^164,165^ Prof. Mohammad Ali Sahraian,^47^ Mehrnoosh Samaei,^166^ Mete Saylan,^167^ Feng Sha,^168^ Masood Ali Shaikh,^169^ Prof. Kenji Shibuya,^170^ Mika Shigematsu,^171^ Prof. Jae Il Shin,^172^ Rahman Shiri,^173^ Prof. Diego Augusto Santos Silva,^174^ Prof. Jasvinder A Singh,^175,176^ Prof. Deepika Singhal,^177,178^ Valentin Yurievich Skryabin,^179^ Anna Aleksandrovna Skryabina,^180^ Amin Soheili,^181^ Houman Sotoudeh,^182^ Emma Elizabeth Spurlock,^1^ Prof. Cassandra E I Szoeke,^183,184^ Prof. Rafael Tabarés-Seisdedos,^185,186^ Biruk Wogayehu Taddele,^187^ Marcos Roberto Tovani-Palone,^188,189^ Gebiyaw Wudie Tsegaye,^190^ Marco Vacante,^191^ Prof. Narayanaswamy Venketasubramanian,^192,193^ Simone Vidale,^194,195^ Prof. Vasily Vlassov,^196^ Giang Thu Vu,^197^ Yuan-Pang Wang,^198^ Jordan Weiss,^199^ Abrha Hailay Weldemariam,^200^ Ronny Westerman,^201^ Prof. Anders Wimo,^202^ Prof. Andrea Sylvia Winkler,^203,204^ Chenkai Wu,^205,206^ Ali Yadollahpour,^207^ Metin Yesiltepe,^208,209^ Naohiro Yonemoto,^210,211^ Prof. Chuanhua Yu,^212^ Prof. Mikhail Sergeevich Zastrozhin,^213,214^ Anasthasia Zastrozhina,^215^ Zhi-Jiang Zhang,^216^ Prof. Christopher J L Murray,^1,5^ and Prof. Theo Vos.^1,5^

^1^Institute for Health Metrics and Evaluation, University of Washington, Seattle, WA, USA; ^2^Department of Neurology, Cairo University, Cairo, Egypt; ^3^Department of Parasitology and Mycology, Jahrom University of Medical Sciences, Jahrom, Iran; ^4^Department of Clinical Sciences, University of Sharjah, Sharjah, United Arab Emirates; ^5^Department of Health Metrics Sciences, School of Medicine, University of Washington, Seattle, WA, USA; ^6^Institute for Advanced Medical Research and Training, University of Ibadan, Ibadan, Nigeria; ^7^Institute of Neuroscience, Newcastle University, Newcastle upon Tyne, UK; ^8^Imam Abdulrahman Bin Faisal University, Dammam, Saudi Arabia; ^9^Health Management and Economics Research Center, Iran University of Medical Sciences, Tehran, Iran; ^10^Health Economics Department, Iran University of Medical Sciences, Tehran, Iran; ^11^Department of Epidemiology, Arak University of Medical Sciences, Arak, Iran; ^12^Students' Scientific Research Center, Tehran University of Medical Sciences, Tehran, Iran; ^13^School of Public Health, Curtin University, Perth, WA, Australia; ^14^Department of Psychiatry, Autonomous University of Madrid (Universidad Autónoma de Madrid), Madrid, Spain; ^15^CIBERSAM, Institute of Health Carlos III, Madrid, Spain; ^16^Unit of Biochemistry, Sultan Zainal Abidin University (Universiti Sultan Zainal Abidin), Kuala Terengganu, Malaysia; ^17^Department of Hypertension, Medical University of Lodz, Lodz, Poland; ^18^Polish Mothers' Memorial Hospital Research Institute, Lodz, Poland; ^19^Department of Neurosciences, Costa Rican Department of Social Security, San Jose, Costa Rica; ^20^School of Medicine, University of Costa Rica, San Pedro, Costa Rica; ^21^School of Psychology, University of Auckland, Auckland, New Zealand; ^22^Department of Psychiatry, University of Münster, Münster, Germany; ^23^Department of Psychiatry, Melbourne Medical School, Melbourne, VIC, Australia; ^24^Social and Clinical Pharmacy, Hradec Kralova, Czech Republic; ^25^Institute of Public Health, United Arab Emirates University, Al Ain, United Arab Emirates; ^26^Department of Statistical and Computational Genomics, National Institute of Biomedical Genomics, Kalyani, India; ^27^Department of Statistics, University of Calcutta, Kolkata, India; ^28^Social Determinants of Health Research Center, Babol University of Medical Sciences, Babol, Iran; ^29^Department of Neurology, Institute of Post-Graduate Medical Education and Research and Seth Sukhlal Karnani Memorial Hospital, Kolkata, India; ^30^Department of Internal Medicine, Manipal Academy of Higher Education, Mangalore, India; ^31^Department of Public Health and Primary Care, University of Cambridge, Cambridge, UK; ^32^Division of Clinical Epidemiology and Aging Research, German Cancer Research Center, Heidelberg, Germany; ^33^Department of Community Medicine, Employee State Insurance Post Graduate Institute of Medical Sciences and Research, Bangalore, India; ^34^Research Unit on Applied Molecular Biosciences (UCIBIO), University of Porto, Porto, Portugal; ^35^Department of Psychiatry, University of Melbourne, Melbourne, VIC, Australia; ^36^National School of Public Health, Institute of Health Carlos III, Madrid, Spain; ^37^Clinical Epidemiology Program, Ottawa Hospital Research Institute, Ottawa, ON, Canada; ^38^Mary MacKillop Institute for Health Research, Australian Catholic University, Melbourne, VIC, Australia; ^39^School of Public Health, University of Hong Kong, Hong Kong, China; ^40^Research School of Population Health, Australian National University, Canberra, ACT, Australia; ^41^Faculty of Biology, Hanoi National University of Education, Hanoi, Vietnam; ^42^Department of Medical Clinic, Federal University of Santa Catarina, Florianópolis, Brazil; ^43^Development of Research and Technology Center, Ministry of Health and Medical Education, Tehran, Iran; ^44^School of Population Health and Environmental Sciences, King's College London, London, UK; ^45^School of Nursing and Midwifery, La Trobe University, Melbourne, VIC, Australia; ^46^Department of Nursing, Umeå University, Umea, Sweden; ^47^Multiple Sclerosis Research Center, Tehran University of Medical Sciences, Tehran, Iran; ^48^Department of Psychology, Federal University of Sergipe, São Cristóvão, Brazil; ^49^Non-communicable Diseases Research Center, Tehran University of Medical Sciences, Tehran, Iran; ^50^National Institute for Stroke and Applied Neurosciences, Auckland University of Technology, Auckland, New Zealand; ^51^Research Center of Neurology, Moscow; ^52^Russia, ; ^53^Department of Neurobiology, Karolinska Institute, Stockholm, Sweden; ^54^Division of Neurology, University of Ottawa, Ottawa, ON, Canada; ^55^Associated Laboratory for Green Chemistry (LAQV), University of Porto, Porto, Portugal; ^56^Research Center on Public Health, University of Milan Bicocca, Monza, Italy; ^57^Psychiatry Department, Kaiser Permanente, Fontana, CA, USA; ^58^School of Health Sciences, A.T. Still University, Mesa, AZ, USA; ^59^Institute of Gerontological Health Services and Nursing Research, Ravensburg-Weingarten University of Applied Sciences, Weingarten, Germany; ^60^Department of Medicine, Datta Meghe Institute of Medical Science, Wardha, India; ^61^Department of Cardiovascular, Endocrine-metabolic Diseases, and Aging, Italian National Health Institute (Istituto Superiore di Sanità (ISS)), Roma, Italy; ^62^Department of Nursing, Aksum University, Aksum, Ethiopia; ^63^Department of Nursing, Mekelle University, Mekelle, Ethiopia; ^64^Student Research Committee, Iran University of Medical Sciences, Tehran, Iran; ^65^Department of Epidemiology and Prevention, IRCCS Neuromed, Pozzilli, Italy; ^66^Third Department of Neurology, Research Center of Neurology, Moscow, Russia; ^67^Health Systems and Policy Research, Indian Institute of Public Health Gandhinagar, Gandhinagar, India; ^68^Department of Preventive Cardiology, Eternal Heart Care Centre & Research Institute, Jaipur, India; ^69^Department of Medicine, Mahatma Gandhi University Medical Sciences, Jaipur, India; ^70^Clinical Neurological Sciences, The University of Western Ontario, London, ON, Canada; ^71^Lawson Health Research Institute, London, ON, Canada; ^72^Department of Social and Public Health, Ohio University, Athens, OH, USA; ^73^Flinders University Rural Health - South Australia, Flinders University, Renmark, SA, Australia; ^74^Medical School, University of Western Australia, Perth, WA, Australia; ^75^Department of Neurology, Sir Charles Gairdner Hospital, Perth, WA, Australia; ^76^Independent Consultant, Santa Clara, CA, USA; ^77^School of Nursing and Midwifery, Tehran University of Medical Sciences, Tehran, Iran; ^78^Department of Urban Planning and Design, University of Hong Kong, Hong Kong, China; ^79^College of Science and Engineering, Hamad Bin Khalifa University, Doha, Qatar; ^80^Department of Occupational Safety and Health, China Medical University, Taichung, Taiwan; ^81^Research Center in Epidemiology and Preventive Medicine (EPIMED), Department of Medicine and Surgery, University of Insubria, Varese, Italy; ^82^Department of Community Medicine, University of Ibadan, Ibadan, Nigeria; ^83^Department of Community Medicine, University College Hospital, Ibadan, Ibadan, Nigeria; ^84^Faculty of Medicine, University of Belgrade, Belgrade, Serbia; ^85^Department of Epidemiology, University of Kragujevac, Kragujevac, Serbia; ^86^Research Institute for Endocrine Sciences, Shahid Beheshti University of Medical Sciences, Tehran, Iran; ^87^Department of Health Services Research, University of Tsukuba, Tsukuba, Japan; ^88^Department of Non-Communicable Disease Epidemiology, London School of Hygiene & Tropical Medicine, London, UK; ^89^Knowledge Translation, Centre for Health Evaluation and Outcome Sciences, Vancouver, BC, Canada; ^90^School of Population and Public Health, University of British Columbia, Vancouver, BC, Canada; ^91^Department of Community Medicine, Dr. Baba Saheb Ambedkar Medical College & Hospital, Delhi, India; ^92^Department of Community Medicine, Banaras Hindu University, Varanasi, India; ^93^Department of Neurology, University of Washington, Seattle, WA, USA; ^94^Institute for Epidemiology and Social Medicine, University of Münster, Münster, Germany; ^95^Department of Adult Health Nursing, Bahir Dar University, Bahir Dar, Ethiopia; ^96^Department of Public Health, Jordan University of Science and Technology, Irbid, Jordan; ^97^Department of Epidemiology and Biostatistics, Health Services Academy, Islamabad, Pakistan; ^98^Global Evidence Synthesis Initiative, Datta Meghe Institute of Medical Sciences, Wardha, India; ^99^School of Traditional Chinese Medicine, Xiamen University Malaysia, Sepang, Malaysia; ^100^Department of Nursing and Health Promotion, Oslo Metropolitan University, Oslo, Norway; ^101^School of Health Sciences, Kristiania University College, Oslo, Norway; ^102^Global Community Health and Behavioral Sciences, Tulane University, New Orleans, LA, USA; ^103^Department of Epidemiology and Public Health, University College London, London, UK; ^104^Department of Public Health, University of Helsinki, Helsinki, Finland; ^105^CIBERSAM, San Juan de Dios Sanitary Park, Sant Boi de Llobregat, Spain; ^106^Catalan Institution for Research and Advanced Studies (ICREA), Barcelona, Spain; ^107^Department of Psychiatry, University of Nairobi, Nairobi, Kenya; ^108^Division of Psychology and Language Sciences, University College London, London, UK; ^109^Unit of Genetics and Public Health, Institute of Medical Sciences, Las Tablas, Panama; ^110^Ministry of Health, Herrera, Panama; ^111^Department of Otorhinolaryngology, Father Muller Medical College, Mangalore, India; ^112^Department of Sociology, Shenzhen University, Shenzhen, China; ^113^Department of Systems, Populations, and Leadership, University of Michigan, Ann Arbor, MI, USA; ^114^Department of Biochemistry, BGS Global Institute of Medical Sciences, Bengaluru, India; ^115^Department of Primary Care and Public Health, Imperial College London, London, UK; ^116^Department of Pediatrics, Montefiore Medical Center, New York, NY, USA; ^117^Department of Environmental Medicine and Public Health, Icahn School of Medicine at Mount Sinai, New York, NY, USA; ^118^Neurology Department, Janakpuri Super Specialty Hospital Society, New Delhi, India; ^119^Department of Neurology, Govind Ballabh Institute of Medical Education and Research, New Delhi, India; ^120^Forensic Medicine Division, Imam Abdulrahman Bin Faisal University, Dammam, Saudi Arabia; ^121^Internal Medicine Department, King Saud University, Riyadh, Saudi Arabia; ^122^Department of Biomolecular Sciences, University of Missippi, Oxford, MS, USA; ^123^Department of Pharmacy, Mizan-Tepi University, Mizan, Ethiopia; ^124^World Health Organization (WHO) Centre on eHealth, University of New South Wales, Sydney, NSW, Australia; ^125^Institute of Epidemiology and Medical Biometry, Ulm University, Ulm, Germany; ^126^Department of Biotechnology, University of Central Punjab, Lahore, Pakistan; ^127^Department of Forensic Medicine and Toxicology, Manipal Academy of Higher Education, Manipal, India; ^128^Institute for Global Health Innovations, Duy Tan University, Hanoi, Vietnam; ^129^Unit of Microbiology and Public Health, Institute of Medical Sciences, Las Tablas, Panama; ^130^Department of Public Health, Ministry of Health, Herrera, Panama; ^131^Department of Psychiatry and Behavioural Neurosciences, McMaster University, Hamilton, ON, Canada; ^132^Department of Psychiatry, University of Lagos, Lagos, Nigeria; ^133^Henry M Jackson School of International Studies, University of Washington, Seattle, WA, USA; ^134^Laboratory of Public Health Indicators Analysis and Health Digitalization, Moscow Institute of Physics and Technology, Dolgoprudny, Russia; ^135^Department of Medicine, University of Ibadan, Ibadan, Nigeria; ^136^Department of Medicine, University College Hospital, Ibadan, Ibadan, Nigeria; ^137^Iran University of Medical Sciences, Tehran, Iran; ^138^Department of Neurology and Public Health, Icahn School of Medicine at Mount Sinai, New York, NY, USA; ^139^Shanghai Mental Health Center, Shanghai Jiao Tong University, Shanghai, China; ^140^Department of Psychiatry, Columbia University, New York City, NY, USA; ^141^Research Center of Neurology, Moscow, Russia; ^142^Discipline of General Practice, University of Newcastle, Callaghan, NSW, Australia; ^143^Department of Pharmacology, Imam Abdulrahman Bin Faisal University, Dammam, Saudi Arabia; ^144^Clinical Research Center, Valle del Lili Foundation (Centro de Investigaciones Clinicas, Fundación Valle del Lili), Cali, Colombia; ^145^PROESA, ICESI University (Centro PROESA, Universidad ICESI), Cali, Colombia; ^146^College of Medicine, University of Central Florida, Orlando, FL, USA; ^147^Thalassemia and Hemoglobinopathy Research Center, Ahvaz Jundishapur University of Medical Sciences, Ahvaz, Iran; ^148^Metabolomics and Genomics Research Center, Tehran University of Medical Sciences, Tehran, Iran; ^149^Department of Public Health, North South University, Dhaka, Bangladesh; ^150^Department of Biostatistics and Epidemiology, University of Massachusetts Amherst, Amherst, MA, USA; ^151^Tehran Institute of Psychiatry, Iran University of Medical Sciences, Tehran, Iran; ^152^Academic Public Health England, Public Health England, London, UK; ^153^WHO Collaborating Centre for Public Health Education and Training, Imperial College London, London, UK; ^154^University College London Hospitals, London, UK; ^155^School of Social Sciences and Psychology, Western Sydney University, Penrith, NSW, Australia; ^156^Translational Health Research Institute, Western Sydney University, Penrith, NSW, Australia; ^157^Research Center for Immunodeficiencies, Tehran University of Medical Sciences, Tehran, Iran; ^158^Network of Immunity in Infection, Malignancy and Autoimmunity (NIIMA), Universal Scientific Education and Research Network (USERN), Tehran, Iran; ^159^Department of Neuroscience, University of Perugia, Perugia, Italy; ^160^Department of Neurology, Rimini "Infermi" Hospital - AUSL Romagna, Rimini, Italy; ^161^Golestan Research Center of Gastroenterology and Hepatology (GRCGH), Golestan University of Medical Sciences, Gorgan, Iran; ^162^School of Psychiatry, University of New South Wales, Kensington, NSW, Australia; ^163^Neuropsychiatric Institute, Prince of Wales Hospital, Randwick, NSW, Australia; ^164^Halal Research Center, Food and Drug Administration of the Islamic Republic of Iran, Tehran, Iran; ^165^Neurogenic Inflammation Research Center, Mashhad University of Medical Sciences, Mashhad, Iran; ^166^Emergency Department, Brown University, Providence, RI, USA; ^167^Market Access, Bayer, Istanbul, Turkey; ^168^Center for Biomedical Information Technology, Shenzhen Institutes of Advanced Technology, Shenzhen, China; ^169^Independent Consultant, Karachi, Pakistan; ^170^Institute for Population Health, King's College London, London, UK; ^171^National Institute of Infectious Diseases, Tokyo, Japan; ^172^College of Medicine, Yonsei University, Seoul, South Korea; ^173^Finnish Institute of Occupational Health, Helsinki, Finland; ^174^Department of Physical Education, Federal University of Santa Catarina, Florianopolis, Brazil; ^175^School of Medicine, University of Alabama at Birmingham, Birmingham, AL, USA; ^176^Medicine Service, US Department of Veterans Affairs (VA), Birmingham, AL, USA; ^177^Department of Ophthalmology, Gmers Medical College and Civil Hospital, Ahmedabad, India; ^178^Department of Ophthalmology, Datta Meghe Institute of Medical Sciences, Wardha, India; ^179^Department No.16, Moscow Research and Practical Centre on Addictions, Moscow, Russia; ^180^Therapeutic Department, Balashiha Central Hospital, Balashikha, Russia; ^181^Nursing Care Research Center, Semnan University of Medical Sciences, Semnan, Iran; ^182^Department of Radiology, University of Alabama at Birmingham, Birmingham, AL, USA; ^183^Faculty of Medicine, Dentistry and Health Sciences, University of Melbourne, Melbourne, VIC, Australia; ^184^The Brain Institute, Australian Healthy Ageing Organisation, Melbourne, VIC, Australia; ^185^Department of Medicine, University of Valencia, Valencia, Spain; ^186^Carlos III Health Institute, Biomedical Research Networking Center for Mental Health Network (CiberSAM), Madrid, Spain; ^187^Department of Pharmacy, Arbaminch College of Health Sciences, Arba Minch, Ethiopia; ^188^Department of Pathology and Legal Medicine, University of São Paulo, Ribeirão Preto, Brazil; ^189^Modestum LTD, London, UK; ^190^College of Medicine and Health Sciences, Bahir Dar University, Bahir Dar, Ethiopia; ^191^Department of General Surgery and Medical-Surgical Specialties, University of Catania, Catania, Italy; ^192^Raffles Neuroscience Centre, Raffles Hospital, Singapore, Singapore; ^193^Yong Loo Lin School of Medicine, National University of Singapore, Singapore, Singapore; ^194^Department of Neurology, Infermi Hospital, Rimini, Italy; ^195^Department of Neurology & Stroke Unit, Sant'Anna Hospital, Como, Italy; ^196^Department of Health Care Administration and Economics, National Research University Higher School of Economics, Moscow, Russia; ^197^Center of Excellence in Behavioral Medicine, Nguyen Tat Thanh University, Ho Chi Minh City, Vietnam; ^198^Department of Psychiatry, University of São Paulo, São Paulo, Brazil; ^199^Department of Demography, University of California Berkeley, Berkley, CA, USA; ^200^Department of Adult Health Nursing, Aksum University, Aksum, Ethiopia; ^201^Competence Center of Mortality-Follow-Up of the German National Cohort, Federal Institute for Population Research, Wiesbaden, Germany; ^202^Department of Neurobiology, Care Sciences and Society, Karolinska Institute, Solna, Sweden; ^203^Institute of Health and Society, University of Oslo, Oslo, Norway; ^204^Department of Neurology, Technical University of Munich, Munich, Germany; ^205^Global Health Research Center, Duke Kunshan University, Kunshan, China; ^206^Duke Global Health Institute, Duke University, Durham, NC, USA; ^207^Psychology Department, University of Sheffield, Sheffield, UK; ^208^Ankara City Hospital, Ankara Provincial Health Directorate, Ankara, Turkey; ^209^Clinical Investigation Unit, Ankara City Hospital, Ankara, Turkey; ^210^Department of Neuropsychopharmacology, National Center of Neurology and Psychiatry, Kodaira, Japan; ^211^Department of Public Health, Juntendo University, Tokyo, Japan; ^212^Department of Epidemiology and Biostatistics, Wuhan University, Wuhan, China; ^213^Laboratory of Genetics and Genomics, Moscow Research and Practical Centre on Addictions, Moscow, Russia; ^214^Addictology Department, Russian Medical Academy of Continuous Professional Education, Moscow, Russia; ^215^Pediatrics Department, Russian Medical Academy of Continuous Professional Education, Moscow, Russia; ^216^School of Medicine, Wuhan University, Wuhan, China.
